# Multi-targeted trehalose-6-phosphate phosphatase I harbors a novel peroxisomal targeting signal 1 and is essential for flowering and development

**DOI:** 10.1007/s00425-020-03389-z

**Published:** 2020-04-18

**Authors:** Amr R. A. Kataya, Ahmed Elshobaky, Behzad Heidari, Nemie-Feyissa Dugassa, Jay J. Thelen, Cathrine Lillo

**Affiliations:** 1 Centre for Organelle Research, Faculty of Science and Technology, University of Stavanger, 4036 Stavanger, Norway; 2 Present Address: Department of Biochemistry, Christopher S. Bond Life Sciences Center, University of Missouri, Columbia, MO USA; 3 Botany Department, Faculty of Science, Mansoura University, Mansoura, 35516 Egypt; 4 Department of Plant Biology, School of Biology, College of Science, University of Tehran, Tehran, Iran

**Keywords:** Abiotic stress, Phosphorylation, Plastid, PTS1, Sucrose

## Abstract

**Main conclusion:**

**This work reveals information about new peroxisomal targeting signals type 1 and identifies trehalose-6-phosphate phosphatase I as multitargeted and is implicated in plant development, reproduction, and stress response.**

**Abstract:**

A putative, non-canonical peroxisomal targeting signal type 1 (PTS1) Pro-Arg-Met > was identified in the extreme C-terminus of trehalose-6-phosphate phosphatase (TPP)I. TPP catalyzes the final step of trehalose synthesis, and the enzyme was previously characterized to be nuclear only (Krasensky et al. in Antioxid Redox Signal 21(9):1289–1304, 2014). Here we show that the TPPI C-terminal decapeptide ending with Pro-Arg-Met > or Pro-Lys-Met > can indeed function as a PTS1. Upon transient expression in two plant expression systems, the free C- or N-terminal end led to the full-length TPPI targeting to peroxisomes and plastids, respectively. The nucleus and nucleolus targeting of the full-length TPPI was observed in both cases. The homozygous T-DNA insertion line of *TPPI* showed a pleiotropic phenotype including smaller leaves, shorter roots, delayed flowering, hypersensitivity to salt, and a sucrose dependent seedling development. Our results identify novel PTS1s, and TPPI as a protein multi-targeted to peroxisomes, plastids, nucleus, and nucleolus. Altogether our findings implicate an essential role for TPPI in development, reproduction, and cell signaling.

**Electronic supplementary material:**

The online version of this article (10.1007/s00425-020-03389-z) contains supplementary material, which is available to authorized users.

## Introduction

Knowledge of the localization of enzymes, metabolites, and regulators is crucial for understanding their cellular function. Subcellular localization of metabolic pathways is one of the principal forms of regulation and is still an ongoing endeavor in plants. Although the subcellular localization of major metabolic pathways is established, bypass or backup in alternative compartments are still being identified (Sweetlove and Fernie 2013). Even the spatial organization of the main metabolic pathways suggests multi-tasking. For example, some enzymes that are part of the oxidative pentose phosphate pathway in cytosol and plastids were recently also found in peroxisomes, where they most likely provide a source of NADPH using sugar phosphates as substrates (Meyer et al. 2011). Peroxisomes have specialized carriers for transport of cofactors such as NAD^+^, ADP, and AMP (Bernhardt et al. 2012), and pore-forming, anion-selective channels facilitate the diffusion of carboxylic acids (Hu et al. 2012). Transport of trehalose and trehalose-6-phosphate (T6P) has to our knowledge not been studied, but it possibly occurs through active transporters as well as by facilitated or regular diffusion, depending upon the organelle. Trehalose has a molecular mass of 342 Da, and trehalose phosphate has a molecular mass of 422 Da. Trehalose, at least, is a candidate to easily diffuse across the peroxisomal membrane since this membrane is assumed to allow free diffusion of molecules up to 300–400 Da and shares a common pool of small metabolites with the cytoplasm (Antonenkov and Hiltunen 2012).

Trehalose is a disaccharide that accumulates and protects cells from drought, salt, or low-temperature stress in most prokaryotic and eukaryotic microorganisms (Crowe et al. 1992; O'Hara et al. 2013). In higher plants, sucrose may have taken over many of the functions of trehalose. With the exceptions known from lower plants, trehalose and T6P are only present in trace amounts in plants. Perturbation of trehalose metabolism does, however, lead to a wide range of different effects in higher plants, such as altered stress tolerance, leaf morphology, and embryo lethality, hence trehalose metabolites are believed to have critical regulatory roles (Vandesteene et al. 2012; Lunn et al. 2014).

T6P is synthesized by trehalose-6-phosphate synthase (TPS) from glucose-6-phosphate and UDP-glucose in the cytosol according to a scheme similar to sucrose-phosphate synthesis (Ruan 2014). Subsequently, T6P is dephosphorylated by trehalose-6-phosphate phosphatase (TPP) to form trehalose. Although higher plants do not accumulate high levels of trehalose, they encode large families of genes (putatively) involved in the synthesis of trehalose, indicating crucial functions of trehalose and trehalose metabolites also in plants. Arabidopsis has eleven genes encoding TPS and ten genes encoding TPP. The TPP genes are named *TPPA-J* (Blazquez et al. 1998; Vandesteene et al. 2012; Lunn et al. 2014), and investigations into TPPI localization and function are presented here.

The primary developmental transition in angiosperms is the switch from vegetative meristem, producing leaves and stem into a floral meristem, producing flowers that complete the plant life cycle. In higher plants, efficient sexual reproduction and ensuring optimal development of seed set and fruits depend on the transition to flowering at an appropriate time. This needs coordinating of flowering time with seasonal and developmental cues (Simpson and Dean 2002; Amasino 2010). Arabidopsis contains one repressor pathway responding to endogenous signals (autonomous inhibition pathway) and six promoting pathways among which three are responding to endogenous signals (autonomous induction pathway, gibberellic acid, and aging pathways) and the other three (vernalization, ambient temperature, and photoperiodic pathways) are responding to exogenous signals (Heidari et al. 2013). The nutritional status of the plant, including sucrose status, is known to be a critical factor in the flowering time regulations (Corbesier et al. 1998; Tsai and Gazzarrini 2014). T6P, as an indicator of sucrose status, plays a vital role in the floral transition. T6P promotes flowering through induction of *FLOWERING LOCUS T *(*FT*) (photoperiodic pathway) in the leaves and *SQUAMOSA PROMOTOR-LIKE *(*SPL*) in the shoot apical meristem (Wahl et al. 2013; Tsai and Gazzarrini 2014).

Localization and transport of trehalose and T6P are still disputed (Ruan 2014), and the enzyme catalyzing the last step of trehalose synthesis, TPP, was recently found to be localized in different subcellular compartments, i.e., cytosol, nucleus/nucleolus, and chloroplasts (Krasensky et al. 2014). Krasensky et al. (2014) demonstrated both TPPD and TPPE are chloroplast localized. The present work shows that TPPI localizes to both plastids and peroxisomes, in both Arabidopsis mesophyll cells and onion epidermal cells. Peroxisomal targeting was demonstrated by in silico studies, and molecular cytological analyses using modified versions of the C-terminal peroxisomal targeting signal. Physiological examination of Arabidopsis *tppi* knockout showed the importance of this gene for flowering time, salt stress, and plant development.

## Materials and methods

### Gene cloning for in planta expression

Arabidopsis *TPPI*, variant 1, cDNAs were amplified from isolated RNA (isolated from roots and flowers) using the primers (F: GCGGCCGCTATGTCAGCTAGTCAAAACAT and R: GAGCTCTCACATTCTTGGCTGCATTT) and (F: ATGAGCTCTCATGTCAGCTAGTCAAAACATTGTCG and R: ATGCGGCCGCCATTCTTGGCTGCATTTGTTTCC) to clone them in the back and front of enhanced yellow fluorescent protein (EYFP), respectively. The C-terminal peroxisomal targeting domain (PTD), comprising C-terminus 10 residues of TPPI, (PRM >) construct was amplified from the EYFP template using (F: CACCATGGCAATGGTGAGCAAGGGCGAGGAG and R: TATGTCTAGAGTCAcattcttggctgcatttgtttccattccacCTTGTACAGCTCGTCCATGCC). The PTD (PKM >) was re-amplified from the PTD (PRM >) construct using the primers (F: CACCATGGCAATGGTGAGCAAGGGCGAGGAG and R: CAAGTCTAGAGTCACATTTTTGGCTGCATTTGT). The deletions of c-terminal two residues were done using the combination with the reverse primer (R: CAAGTCTAGAGTCATGGCTGCATTTGTTTCCATTC) for both the full-length cDNA and the PTD. The deletion of N-terminal 10 residues was done using the combination with the reverse primer (ATGAGCTCTCATGGAGACTACAATGTCAAGTATCATC). The cDNAs were cloned in pGEMT-Easy, and subsequently subcloned into pCAT-EYFP vector (Ma et al. 2006; Ma and Reumann 2008) to create N-terminal or C-terminal protein fusions with EYFP. All the subcloning vectors are under the control of a 35S promoter of cauliflower mosaic virus.

### RT-PCR

Total RNA was extracted using the RNeasy Plant Mini Kit (Qiagen) according to the manufacturer’s protocol. First-strand cDNA synthesis was performed using Superscript III reverse transcriptase (Invitrogen) in a 20-µl standard reaction mixture containing gene-specific primers. PCR amplification was done using the Expand High Fidelity^PLUS^ PCR System (Roche), using 200 ng RNA equivalents of cDNA per reaction. The primers used for expression analysis of TPPI are F: TCATGTCAAGCAAGATGAGAAGAACAGT that is spanning exon 2 and 3, and R: TCGACGCAGCGAAAGTGCAC that is located in exon 6.

### Bioinformatics

Sequencing of the recombinant constructs was accomplished by Microsynth Seqlab GmbH (Göttingen, Germany) using their facility of Extended Hotshots reactions. The general promoters T7, SP6, 35S, and NOS terminator primers were used for sequencing in pGEM-T Easy and EYFP-containing plasmids. Sequence analysis was done using Vector NTI (Invitrogen) in combination with web-based programs for reversing DNA (https://www.bioinformatics.org/SMS/rev_comp.html) and protein translation (https://us.expasy.org/tools/dna.html). The prediction site used for predicting peroxisomal targeting signal type 1 (PTS1) scores is PredPlantPTS1, https://ppp.gobics.de/ (Lingner et al. 2011; Reumann et al. 2012). Phylogenetic relationships were inferred by preferential alignments of the protein sequences obtained from NCBI. This was done using the program MEGA6 (Tamura et al. 2013) and vector NTI (Invitrogen).

### Transformation and microscopy

Arabidopsis (Col-0) WT seeds were sown on soil and stratified for two days, then transferred to 12 h light/12 h dark conditions, and irrigated once a week by complete Hoagland solution. Plants three- to four-week-old were used for protoplast isolation. For transformation analysis in Arabidopsis mesophyll protoplasts, amplified constructs, and peroxisomal marker proteins were co-transformed into protoplasts using polyethylene glycol transformation protocols (Yoo et al. 2007). For transformation analysis in onion epidermal cells and leaves, plasmids were transiently introduced by a helium-driven particle accelerator (PDS/1000; Bio-Rad) with all necessary adjustments set according to the manufacturer’s recommendations. The bombarded tissues were incubated for one to two days in the dark at room temperature and then observed under the microscope. Four-week-old tobacco leaves were used for transient transformation of EYFP-TPPI by agroinfiltration, and confocal imaging was performed as described previously (Kataya et al. 2016).

Peroxisomal markers used were gMDH-CFP (Fulda et al. 2002) that contains 50 N-terminal amino acids (including the peroxisomal targeting signal type 2 (PTS2): a nonapeptide with a prototype Arg-Leu-(X)5-His-Leu located at the N-termini of proteins) from *Cucumis sativus* glyoxysomal malate dehydrogenase linked with a cyan fluorescent protein (Kim and Smith 1994). Microscopy was carried out using a Nikon TE-2000U inverted fluorescence microscope equipped with an Exfo X-Cite 120 fluorescence illumination system and filters for CFP (exciter S436/10, emitter S470/30), YFP (exciter HQ500/20, emitter S535/30), Texas red filter set for RFP or OFP: 31,004 (exciter D560/40 × , emitter D630/60 m), and a particular red chlorophyll autofluorescence filter (exciter HQ630/39, emitter HQ680/40; Chroma Technologies). Images were captured using a Hamamatsu Orca ER 1394 cooled CCD camera. Images were subsequently processed for optimal presentation with Adobe Photoshop version 9.0.

### Plant growth

Arabidopsis mutant lines for the TPPI (SAIL-354-D09 (*tppi*), SAIL_1245_A06, and GK-480G12) were obtained from the European Arabidopsis Stock Centre (Nottingham, UK). Mutant selection was done by PCR using primers (SAIL_354_D09-LP: TTCAATCATTGGACGGATTTC, SAIL_354_D09-RP: ACGACAGATGCAACATCCTTC, SAIL_1245_A06_LP: TTCAATCATTGGACGGATTTC, SAIL_1245_A06_RP: ACGACAGATGCAACATCCTTC, GABI_480G12_LP: CAATGCATTCATAATCTGTGGG, GABI_480G12_RP: AGACGAACCTTGCTTGACATG) for T-DNA insertion lines recommended at the SALK institute website SIGnAL (https://www.signal.salk.edu/tdnaprimers.2.html). The *pex14* mutant (Orth et al. 2007; Zhang and Hu 2010) seeds were kindly provided by Prof. Jianping Hu, MSU, USA. Wild type (WT) Arabidopsis seeds were obtained from the Arabidopsis Biological Resource Center (ABRC, Columbus, Ohio, USA). For plant material grown on soil, seeds were sown directly in a regular soil–plant mix. Seeds were stratified at 4 °C for two days and then transferred to standard growth conditions. During germination and growth, plants were placed at 22 °C under artificial light in short days 8 h light/16 h dark, 12 h light/12 h dark, or long days 16 h light/8 h dark regimens.

### Generation of transgenic lines

To generate an overexpressor line of N-terminally fused protein, gene-specific primers were used to amplify full-length Arabidopsis cDNA of TPPI, and cloned in the pGEMT-EYFP vector using the primers (F: aaTTAATTAACATGTCAGCTAGTCAAAACATTGTC and R: aaACTAGTTCACATTCTTGGCTGCATTTGTTT). Subsequently, the available EYFP-TPPI was excised and subcloned into the pBA002 vector. The construct was transformed into *A. tumefaciens* strain *ABI-1* via the freeze–thaw method. *A. thaliana* Col-0 was transformed by the floral dip method (Clough and Bent 1998). Seeds were screened on half MS agar plates containing 10 µg ml^−1^ phosphinotricin (PPT). PPT resistant seedlings were selected 10 to 14 days after germination. The successful transformation was validated by isolation of the genomic DNA of the primary transformants and using primers upstream (forward) and downstream (reverse) of the insert.

### Sugar dependence and 2,4-DB, IBA, and OPDA response assays

For sugar dependence analysis, seeds of WT Col-0 and mutant were sown on ½ Linsmeier and Skoog (LS) medium with vitamins (LS; Caisson Labs, Smithfield, UT, USA) with or without 1% (w/v) sucrose and stratified in the dark at 4 °C for two days before being transferred to darkness or short-day conditions (8 h light/16 h dark) at 20 °C. Six-day-old seedlings were scanned using a CANON scanner, and hypocotyls length were measured using ImageJ (Schneider et al. 2012) (https://rsb.info.nih.gov/ij/). To study response to protoauxins, 2,4-DB (2,4-dichlorophenoxyacetic acid), IBA (indole-3-butyric acid), or OPDA (proto-methyl-jasmonic acid) were added to ½ LS agar medium with 1% (w/v) sucrose. Methyl-jasmonic (MeJA) acid was used as a control for OPDA conversion. Seeds were sown and stratified for two days then kept in continuous light for six to seven days. The length of the primary root was measured using ImageJ (Zolman et al. 2001; Zhang and Hu 2010).

### Salt stress experiments

Surface-sterilized seeds were allowed to grow on half-strength LS agar media with 1% sucrose for 4 days in 16 h:8 h of light: dark. Afterward, seedlings were transferred to LS containing 0, 50, 100 or 150 mM salt (NaCl or KCl) and allowed to grow for another 6 or 12 days. The *stt3a-2* T-DNA insertion mutant (ecotype Col-0) was described by Koiwa et al. (2003). The seedlings of *stt3a-2* are hypersensitive to NaCl, KCl, and mannitol (Koiwa et al. 2003). The length of the primary root was measured using ImageJ.

### Flowering time phenotyping

Plants were observed daily. The number of rosette leaves and flowering time were recorded. Flowering time was measured using the appearance of the first open flower as an indicator of transition from inflorescence meristem to floral meristem (Heidari et al. 2013). Characterizing of flowering phenotypes was repeated at least three times and in successive generations, for the *tppi* mutant to assure that observations are repeatable, and phenotype is stable during generations.

## Results

### TPPI has conserved PTS1-like tripeptides

**Fig. 1:**
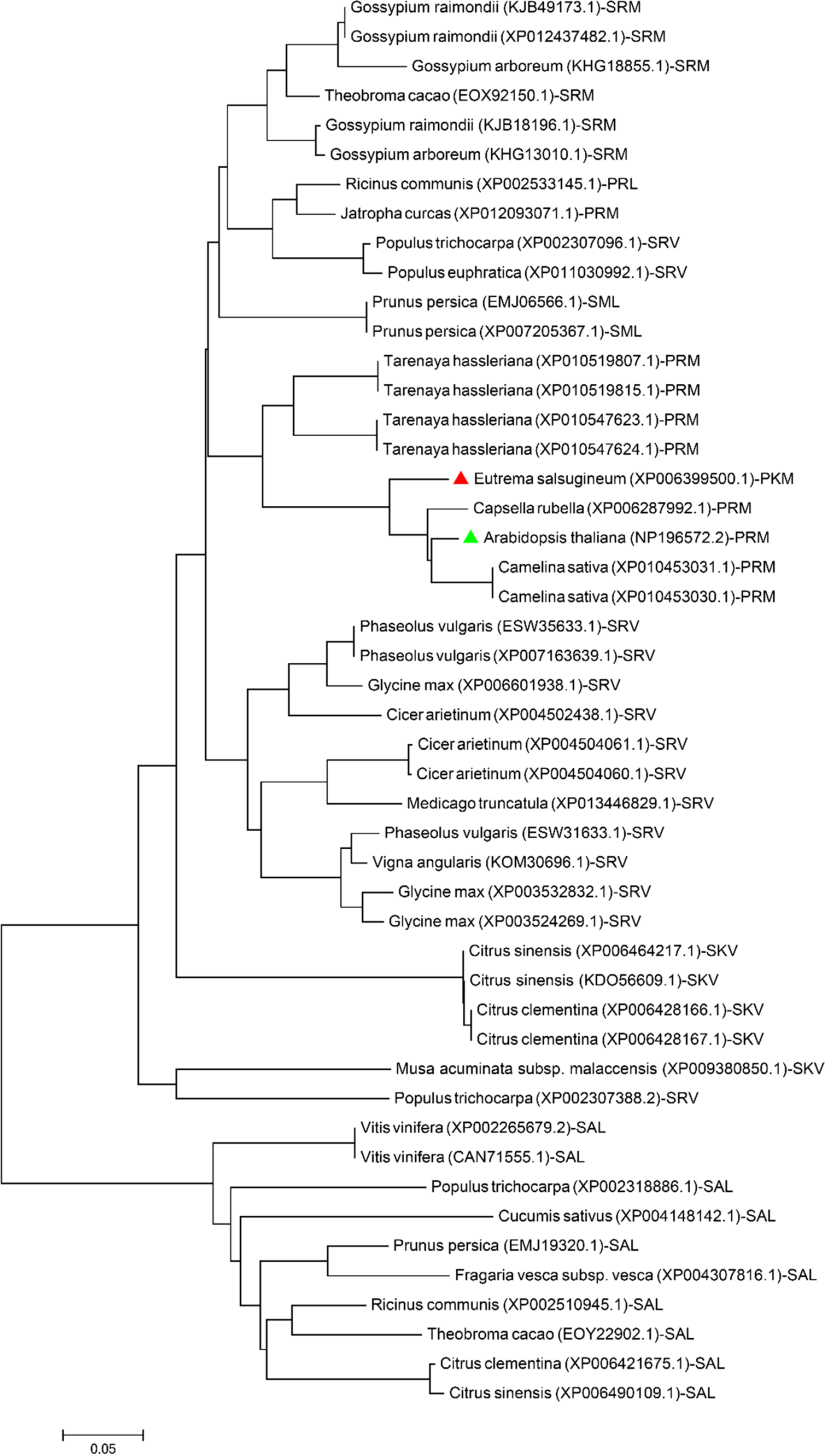
Evolutionary relationship of full-length TPPI homologs that harbors a conserved PTS1. The tripeptide PRM > (highlighted with a green triangle) is conserved in flowering plants. However, a change of PRM > to PKM > (highlighted with a red triangle) in *Eutrema salsugineum* from the family/tribe Brassicaceae/Camelineae also gave a high prediction to be a functional peroxisomal signal. Nevertheless, a conserved PTS1 represented by other known PTS1s was found in the TPPI homologs. The phylogram was generated by MEGA6 (Tamura et al. 2013) using the Neighbor-Joining method of Saitou and Nei. The tree is drawn with branch lengths in the same units as those of the evolutionary distances used to infer the phylogenetic tree. The evolutionary distances were computed using the Poisson correction method and are in the units of the number of amino acid substitutions per site

The major targeting signal that is responsible for the localization of nuclear-encoded peroxisomal proteins is the PTS1 (Reumann 2004). Identifying low-abundant peroxisomal proteins depends mostly on bioinformatic predictions and discovering the non-canonical, rare PTS1s (Reumann 2004; Lingner et al. 2011; Wang et al. 2017; Reumann and Chowdhary 2018). Using recent prediction methods (Lingner et al. 2011; Wang et al. 2017), we identified a putative signal comprising of the tripeptide PRM > in the C-terminus of Arabidopsis TPPI. The PTS1 prediction score for the TPPI sequence was 0.618 and is above the threshold 0.412 (the min./max. score for Arabidopsis is 1.966/1.188, https://ppp.gobics.de) (Lingner et al. 2011; Reumann et al. 2012). Interestingly, PRM > was not previously described as a PTS1 and seems to be a non-canonical signal because it is only present at the C-terminus of TPPI in all of the Arabidopsis proteome (Fig. 1).

Using the BLAST search tool (protein BLAST, NCBI), several homologs of TPPI from only flowering plants showed conservation of known functional PTS1s (such as SRM > , PRL > , SRV > , SKV > , SAL >), and the putative PRM > . Additionally, another putative PTS1 PKM > was located at the TPPI homolog from *Eutrema salsugineum* (Fig. 1, Supplementary Fig. S1). The prediction score for Eutrema TPPI sequence was found to be 6.06, which indicates the functionality of its C-terminal tripeptide as a PTS1. To sum up, these data indicate that the TPPI has functional peroxisomal domains that terminate with PRM > in Arabidopsis and PKM > in Eutrema.

### The tripeptides PRM > and PKM > are novel plant PTS1s

**Fig. 2:**
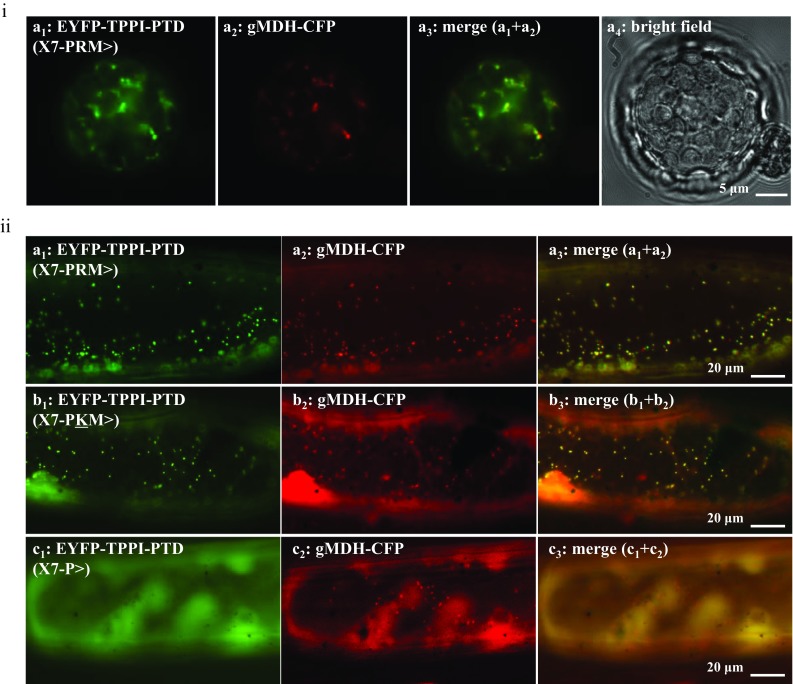
*S*ubcellular localization analysis of TPPI PTDs. To investigate the functionality of the tripeptide PRM > and PKM > as a PTS1, EYFP was extended C-terminally by TPPI PTD comprising the C-terminus 10 residues (Arabidopsis: VEWKQMQPRM, **ia**, **iia**) and (Eutrema: VEWKQMQPKM, **iib**). The extended reporter proteins targeted peroxisomes during transient expression in **i** Arabidopsis mesophyll protoplasts upon polyethylene glycol-mediated transformation or in **ii** onion epidermal cells upon biolistic bombardment. Additionally, two residues were removed from the TPPI EYFP-PTD **iic**, and the fusion protein remained in the cytosol and approved the importance of RM > or KM > for the functionality of the PTS1 signal. Peroxisomes were labeled with gMDH-CFP (Fulda et al. 2002), and the cyan fluorescence was converted to red

To test the functionality of the PRM > as a peroxisomal targeting signal, we fused the putative domain comprising the C-terminal ten residues of Arabidopsis TPPI to EYFP. The extended fusion protein targeted organelle-like structures that coincided with labeled peroxisomes, when expressed in Arabidopsis mesophyll protoplasts (Fig. 2i) and onion epidermal cells (Fig. 2iia). In both plant expression systems, targeting efficiency was high with little fluorescence in the cytosol indicating that PRM > is a strong PTS1. We were also eager to investigate the functionality of Eutrema TPPI domain. Because it harbors the same peptide sequence as Arabidopsis apart from the single residue change (PKM > instead of PRM >), we mutated the extended fluorescent protein (EYFP-X7-PRM >) to (EYFP-X7-PKM >). Similarly, the newly mutated fusion targeted organelle-like structures that coincided with labeled peroxisomes (Fig. 2iib). We also deleted the C-terminal two residues from the extended protein to investigate the importance of RM > or KM > for the targeting pattern (Fig. 2iic). As expected, the modified fluorescent protein remained in the cytosol and failed to target peroxisomes. Taken together, these data show the functionality of TPPI domains from both Arabidopsis and Eutrema and show the functionality of PRM > and PKM > as novel PTS1s.

### Arabidopsis TPPI targets peroxisomes, nucleus, and nucleolus

**Fig. 3:**
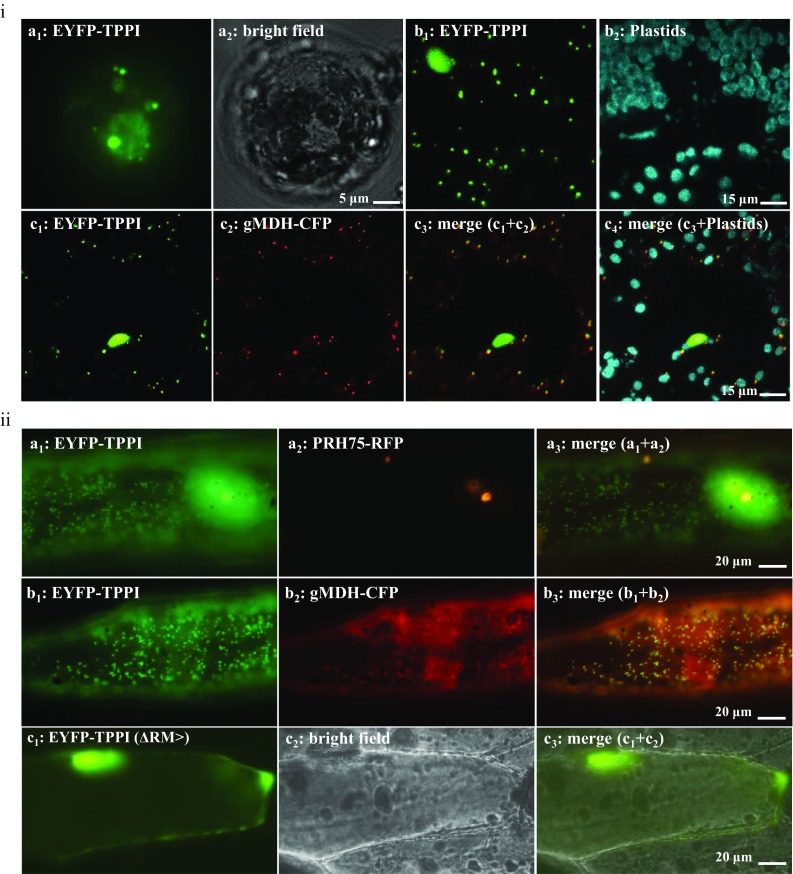
Full-length TPPI with a free C-terminus targets peroxisomes. Arabidopsis TPPI was fused N-terminally with EYFP to form EYFP::TPPI that was expressed transiently in **ia** Arabidopsis mesophyll protoplasts upon polyethylene glycol-mediated transformation, **ib**-**c** in tobacco leaves by agroinfiltration or **ii** onion epidermal cells upon biolistic bombardment. **i** The fusion protein was observed in the nucleus and nucleolus, and punctate structures **a**–**c** that co-localized with labeled peroxisomes **c**. **ii** The fusion protein was observed in nucleus, nucleolus **a**, and peroxisomes **a**, **b**. Moreover, two residues were removed from the EYFP::TPPI **iic**, and the fusion protein did not target peroxisomes and instead targeted nucleus and nucleolus, and decidedly little cytosol. Peroxisomes and nucleolus were labeled with gMDH-CFP and PRH75-RFP (in orange color **iia**_**2**_), respectively. The cyan fluorescence was converted to red. **ib**, **ic** Stacks of 25–30 images captured in Z-stack

To investigate the peroxisomal targeting of the full-length TPPI by PRM > , we amplified the cDNA from RNA that was isolated from flowers and roots. The full-length cDNA was then fused N-terminally with EYFP. The fusion protein was further expressed in Arabidopsis mesophyll protoplasts and tobacco leaves where it was mostly found in the nucleus and nucleolus (Fig. 3ia, ib). The fusion protein was also localized in organelle-like structures that resemble peroxisomes (Fig. 3ia, ib). Subsequently, the full-length fusion protein was expressed simultaneously with a peroxisomal marker and confirmed the targeting of the fusion protein to peroxisomes, as well as nucleus and nucleolus (Fig. 3ic). We also noticed the multitargeting of the fusion protein in onion epidermal cells where the fusion protein targeted nucleus, the labeled nucleolus (Fig. 3iia), and labeled peroxisomes (Fig. 3iib). To confirm that the full-length peroxisomal targeting is PTS1 (PRM >)-dependent, we deleted the c-terminal RM > and further fused the truncated TPPI N-terminally with EYFP. The truncated fusion protein failed to target peroxisomes and localized to the nucleus (Fig. 3iic).

### Arabidopsis TPPI targets plastids

**Fig. 4:**
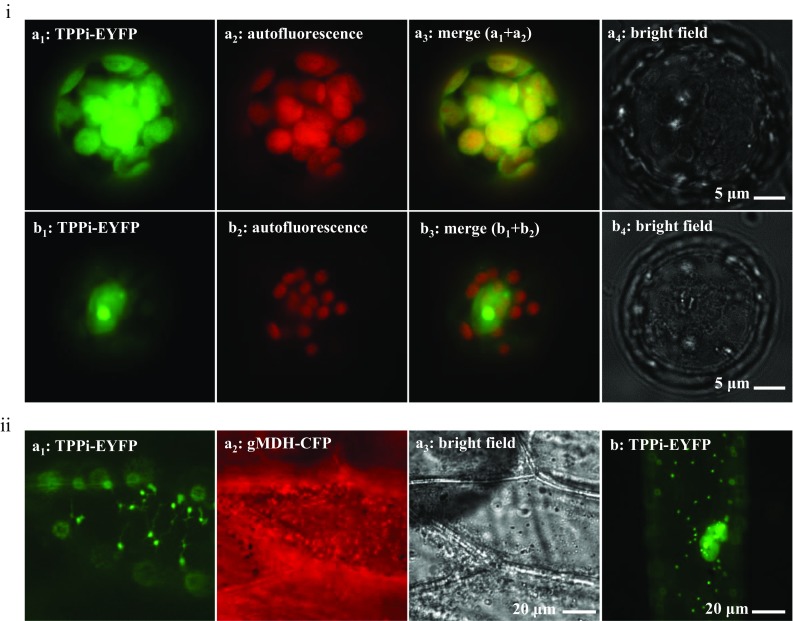
Full-length TPPI with a free N-terminus targets plastids. Arabidopsis TPPI was fused C-terminally with EYFP to form TPPI::EYFP that was expressed transiently in **i** Arabidopsis mesophyll protoplasts and **ii** onion epidermal cells. The fusion protein targeted plastids-like structures in **ia** protoplasts, as well as **ib** nucleus and nucleolus. Fitting with targeting in protoplasts, the fusion protein targeted **iia**, **iib** leucoplasts-like structures, as well as **iib** nucleus and nucleolus in onion epidermal cells. Chloroplasts were captured by chlorophyll autofluorescence. Peroxisomes were labeled with gMDH-CFP, and the cyan fluorescence was converted to red

TPPI is highly predicted to also target plastids by ChloroP 1.1 server (Emanuelsson et al. 1999) with a score of 0.512 and a cleavage site at N-9. To investigate this prediction, we fused the full-length TPPI C-terminally with EYFP and investigated its subcellular localization. The fusion protein was found to target chloroplasts in Arabidopsis mesophyll protoplast (Fig. 4ia). Additionally, nucleus and nucleolus targeting were also noticeable (Fig. 4ib). Besides, we also confirmed its targeting in onion epidermal cells that lack photosynthetic pigments. As expected, the fusion protein targeted leucoplasts (Fig. 4iia) with its clear stromules (known stroma-filled tubules that extend from the surface of all plastid types (Natesan et al. 2005). Multitargeting of the fusion protein to leucoplasts, nucleus, and nucleolus was also observed (Fig. 4iib). We also deleted the N-terminal 10 residues and replaced them with methionine. The subsequent fusion protein was also targeted to plastids in onion epidermal cells (data not shown). We subjected the protein sequence without the N-terminal residues to another prediction server [TargetP 1.1 (Emanuelsson et al. 2000)] that determined a second plastid signal with a cleavage site of 30. Taken together, these data and predictions show the plastid targeting of TPPI.

### The *tppi* mutant seedlings show a sugar dependent phenotype in short days

**Fig. 5:**
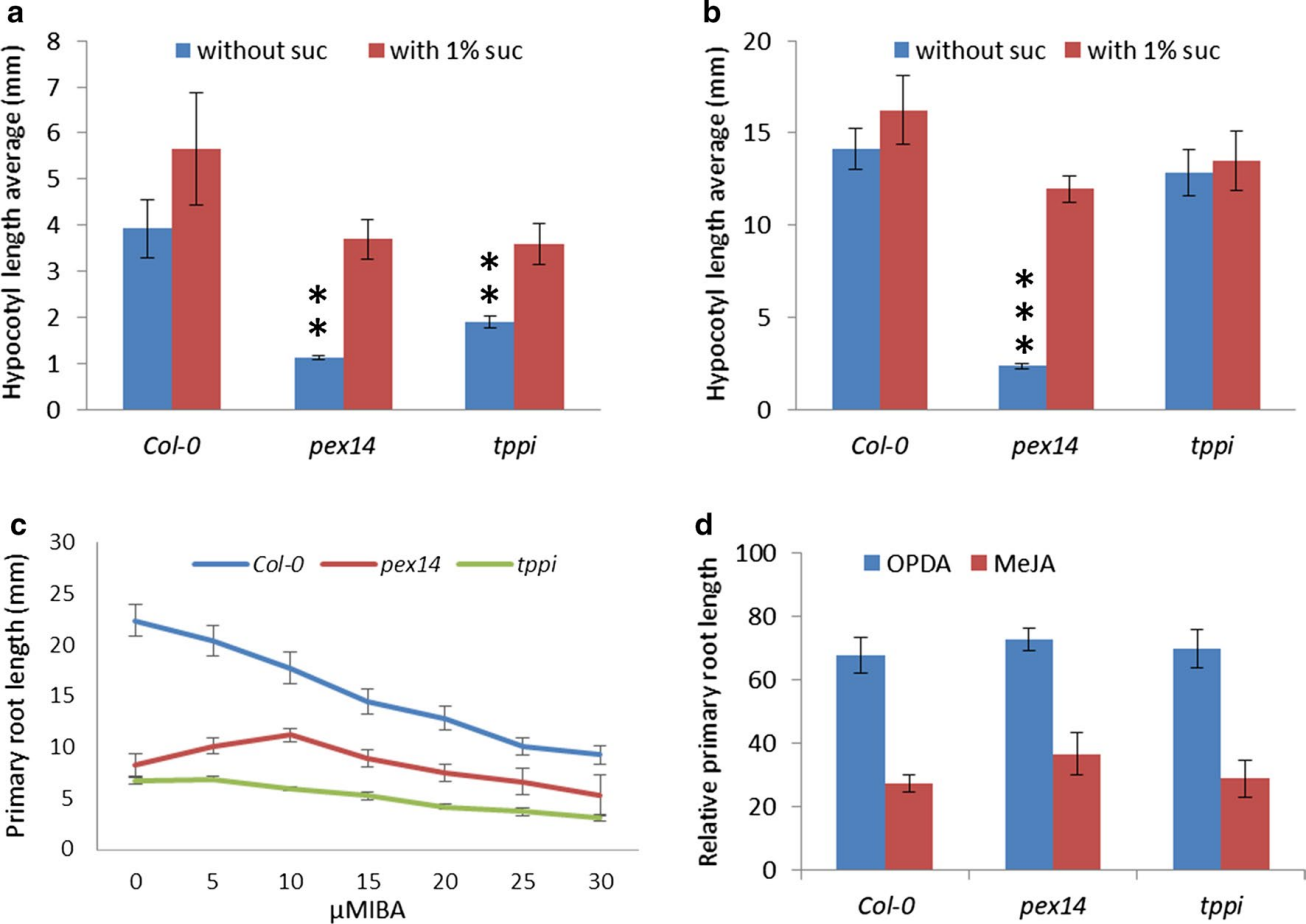
The effect of *TPPI* knockout on seedlings. **a**, **b** Sucrose dependence assay of *tppi* mutant. Hypocotyl length of seedlings grown **a** for 6 d in 8 h light/16 h dark or **b** in the dark on one-half-strength Linsmeier and Skoog (LS) medium with or without 1% Suc. The experiments were repeated three times; error bars represent SE. Columns marked with stars indicate a significant difference between no sucrose and 1% sucrose treatment in Student’s *t* test. Two stars *P* < 0.01, and 3 stars *P* < 0.001. **c**, **d** Effects of IBA and OPDA on primary root elongation of *tppi* mutant seedlings. Relative root lengths (treated versus untreated) of 7-day-old seedlings grown on medium supplemented with 250 nM OPDA or 10 µM methyl-jasmonic (MeJA) acid are shown in **d**. Plants were grown for 7 days in light on one-half-strength LS medium supplemented with 0.5% Suc and different concentrations of **c** IBA and **d** OPDA. The experiments were repeated three times; error bars represent SE

Fatty acid degradation is essential for early seedling development, and seedling growth is halted in mutants defective in degrading fatty acids unless another energy source (ex. sucrose) is provided (Mano and Nishimura 2005; Cassin-Ross and Hu 2014). Homozygous mutants of T-DNA insertion lines were isolated by genotyping for *TPPI* (Supplementary Fig. S2a). Using expression analysis, *TPPI* was found to be knocked out in the *tppi* mutant seedlings (Supplementary Fig. S2b). Subsequently, the *tppi* mutant was used to study the effects of sucrose. The *pex14* mutant that is deficient in the peroxisomal membrane protein PEX14 was used as a sugar dependence control (Orth et al. 2007; Zhang and Hu 2010). In short days, on sucrose-free medium, hypocotyl elongation was strongly inhibited in *pex14* and *tppi* seedlings relative to sucrose-containing medium, as compared with WT (Fig. 5a and Supplementary Fig. S3). The sugar dependence was not apparent for *tppi* seedlings in continuous darkness (Fig. 5b). We also investigated the effects of the proto-auxins (IBA, 2,4-DB) and OPDA, which are processed in peroxisomes (Zolman et al. 2001; Mano and Nishimura 2005; Cassin-Ross and Hu 2014). The *tppi* mutant did not show IBA resistance (Fig. 5c) and responded similarly to WT for the OPDA treatment (Fig. 5d), hence apparently it is not impaired in peroxisomal metabolism of these compounds. The mutant plants show an apparent phenotype when grown on soil, where they show a relatively slower growth as seen after two-to-eight weeks (Supplementary Fig. S4).

### The *tppi* mutant seedlings are hypersensitive to salt stress

The importance of trehalose metabolism in plant stress tolerance is well documented (Vandesteene et al. 2012; Delorge et al. 2014). In Arabidopsis, the *tppf* knockout mutant has decreased tolerance to drought (Lin et al. 2019), and *tppd* is hypersensitive to high-salt stress (Krasensky et al. 2014). In that regard, we investigated the role of TPPI in response to abiotic stress. WT seedlings were germinated on standard nutrient medium and then transferred to medium containing NaCl. The *tppi* seedlings showed hypersensitivity to higher salt concentrations represented by the reduced root growth and higher death rate in 100 mM NaCl (Supplementary Fig. S5), and when treated with KCl (Supplementary Fig. S6). The *tppi* seedlings responses to high salt were comparable to the salt-hypersensitive *stt3a* mutant which was incorporated as a control (Supplementary Fig. S6) (Koiwa et al. 2003).

We generated *TPPI* overexpressor (OEX) and complementation plants. *TPPI* OEX lines were produced by transforming a plasmid coding for either TPPI or EYFP-TPPI fusion into WT plants. To rescue the *TPPI* knockout, the *tppi* mutant was transformed with a construct containing the *TPPI* CDS. All OEX and complementation lines were proved to have higher expression in leaves than WT (data not shown). The *tppi* + 35S-TPPI complementation line was unable to rescue the salt-stress phenotype, and the two OEX lines showed a similar NaCl response as WT (data not shown). In addition, the *tppi* mutant showed similar tolerance as WT to chloroplast oxidative stress induced by methyl viologen and osmotic mannitol-induced stress (data not shown). In conclusion, TPPI seems to play a role in the salt stress response but does not appear to be essential for oxidative and osmotic stress.

### The *tppi* mutant plants display late-flowering phenotype

**Fig. 6:**
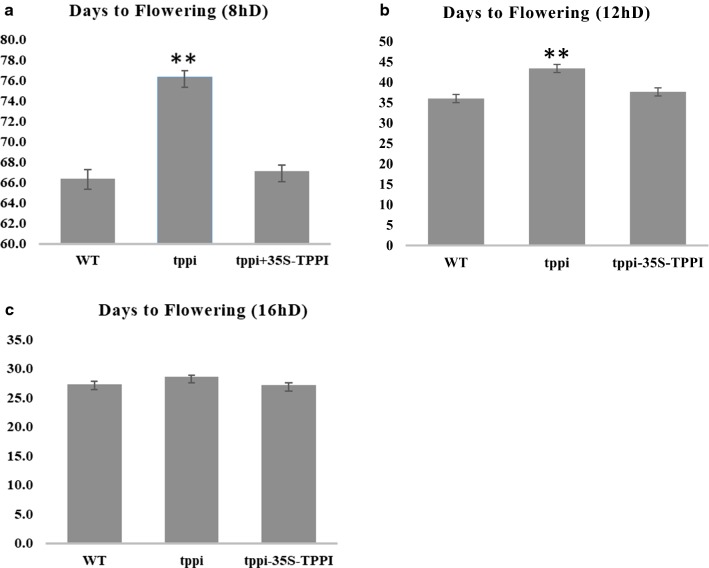
Flowering time phenotyping. **a**–**c** Flowering time for WT, *tppi* and *tppi* + 35S-TPPI plants grown in 8 h day, 12 h day, and 16 h day was calculated based on the appearance of the first flower. Columns marked with two stars are significantly different from WT at *P* < 0.01, (Student’s *t* test)

**Fig. 7:**
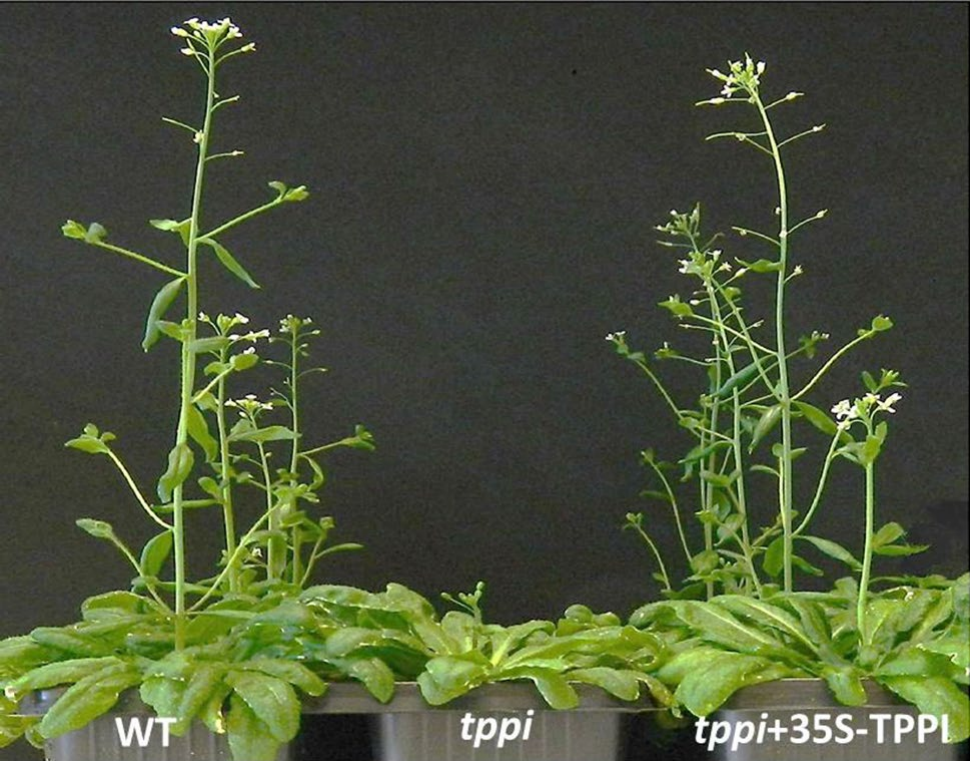
Visible phenotypes of representative *tppi* mutants. Representative plants of WT, *tppi,* and *tppi* + 35S-TPPI. Plants were grown in 8 h days

The *tppi* plants also showed delayed flowering, and the *TPPI* expression is specifically higher in flowers and roots (data not shown), which aligns with the available microarray experiments (Supplementary Fig. S7). That said, plants were grown to test the number of days to flowering. The mutant plants *tppi* and *tppi* + 35S-TPPI were observed in 8 h days, 12 h days, and 16 h days and showed that the first flower appeared later in *tppi* than in WT in both 8 h days and 12 h days (Fig. 6a–c). In the *tppi* mutant, the first flower appeared 10 and 7 days after WT in 8 and 12 days, respectively. The *tppi* + 35S-TPPI plants showed flowering time very similar to WT under all conditions (Fig. 6). The late-flowering phenotype of *tppi* mutant is illustrated in Fig. 7. These experiments implicate a role for TPPI in flowering and verify the ability of 35S-TPPI to complement the flowering phenotype.

## Discussion

In this study, we identified a new, non-canonical but also strong PTS1 (PRM >) at the extreme C-terminus of Arabidopsis TPPI which aligns with the predicted plant PTS1 motif ([SAPC][RKNMSLH][LMIVY] >) (Lingner et al. 2011). In silico search for homologs of TPPI harboring a PTS1 showed that such a signal is widespread in higher plants (Fig. 1). Changing PRM > to PKM > , which is found in Eutrema’s TPPI (Fig. 1; a close Arabidopsis ortholog), helped the verification of the functionality, to our knowledge, of a new PTS1. The N-terminally fused full-length TPPI targeted nucleus/nucleolus, and peroxisomes. Our data agrees partially with Krasensky et al. (2014), where the nucleus and nucleolus only were reported for TPPI localization. Krasensky et al. (2014) could not report the peroxisomal targeting because they masked TPPI-C-terminus PRM > in the C-terminally fused TPPI. The utilization of two plant systems allowed us to verify the ability of the C-terminally fused TPPI to target plastids. Interestingly, TPPI possessed a redox-sensitive cysteine as also found in TPPD and TPPE (Krasensky et al. 2014) and may be redox-regulated by the thioredoxin system in plastids (Lillo 2008; Meyer et al. 2009).

Trehalose metabolites, especially T6P, clearly have a strong influence on plant growth and development (Schluepmann and Paul 2009). *TPS1* expression was necessary to obtain normal root growth. Roots of 8-day-old seedlings grown on a medium, both with and without sucrose were almost wholly inhibited unless TPS1 was induced by dexamethasone (Van Dijken et al. 2004), indicating that the precursor of T6P and/or trehalose are essential for root growth. TPPI is expressed primarily in roots and leaf primordial (Vandesteene et al. 2012; Van Houtte et al. 2013). In support of these general roles for TPP, the *tppi* mutant shows multiple phenotypes such as aberrations in root growth, plant growth, and development.

Mutation of the *TPPI* had significant effects on levels of starch and sucrose, apparently more effective than any of the other *TPP*s (Vandesteene et al. 2012). In this study, we also find *tppi* seedlings to exhibit sucrose-dependence phenotype during early germination. At the same time, decreased TPPI activity should lead to higher levels of T6P, which acts as a signaling molecule in metabolic regulation (Paul 2008; Vandesteene et al. 2012). It has been shown that some effects of T6P may occur through its inhibitory effect on the SNF1-related protein kinase 1 (SnRK1) kinase, which belongs to the SNF1/AMPK group of kinases (Schluepmann and Paul 2009; Zhang et al. 2009; Nuccio et al. 2015). In plants, SnRK1 is involved in the regulation of primary metabolism by inhibiting critical enzymes in sucrose synthesis, nitrogen assimilation and isoprenoid synthesis (Lillo 2008; Wurzinger et al. 2018). SnRK1 inhibits carbohydrate synthesis (sucrose-phosphate synthetase, SPS), nitrogen assimilation (nitrate reductase, NR), and isoprenoid synthesis (3-hydroxy-3-methylglutaryl CoA reductase, HMGR). Inhibition of SnRK1 by T6P will, therefore, support activation of these enzymes, and promote primary (anabolic) metabolism. These enzymes are located in the cytosol, and to our knowledge, it is not known if or how regulation by T6P would be relevant in peroxisomes or chloroplasts. Future studies utilizing a complemented *tppi* mutant with TPPI that lacks a PTS1 could help us get more insights into the function of TPPI targeting and trehalose metabolites in peroxisomes.

*TPPI* is highly expressed in roots and flowers and the GUS-stained seedlings show downregulation of its expression in root tips upon light and sucrose absence (Vandesteene et al. 2012; Van Houtte et al. 2013). Increased tolerance to abiotic stress has, however, previously been observed in plants overexpressing *TPP* or *TPS* genes (Van Dijken et al. 2004; Delorge et al. 2014). Since the role of TPP enzymes is to convert T6P into trehalose, reduced *TPPI* expression would lead to reduced trehalose levels in salt-stressed Arabidopsis. Trehalose is a stress protectant (Elbein et al. 2003) and its metabolism has a critical role in plant stress tolerance (Schluepmann et al. 2003; Delorge et al. 2014). The salt stress sensitivity of *tppi* raises the question if this is a result of the trehalose levels aberration.

Previously, it has been shown that T6P regulates flowering time where a reduction in T6P level delays flowering, while an increase in T6P level promotes flowering (Schluepmann et al. 2003; Wahl et al. 2013). Loss of *TPS1* causes extremely late flowering time in Arabidopsis and overexpression of *TPS1* results in a very early flowering phenotype (Wahl et al. 2013). Here we found that mutation in the *TPPI* gene delays flowering time in 12 h days and under noninductive (8 h days) condition. This shows that for the regulation of flowering time in Arabidopsis, it is probable that not only T6P is sensed but also downstream products of the T6P pathway; for example, trehalose, are also measured. Complementation of *tppi* mutant with 35S-TPPI can rescue the late flowering, which proves that the observed late-flowering phenotype is caused by the mutation in the *TPPI* gene.

## Conclusion

Arabidopsis TPPI, a member of the TPP family, can target multiple subcellular organelles and harbors a novel PTS1. The knockout mutant shows multiple phenotypes, including flowering delay, and emphasizes the complexity and redundancy between the TPP family members.

### *Author contribution statement*

ARAK designed the research; ARAK, BH, AE, and ND performed the experiments; ARAK, BH, and AE analyzed the data; ARAK, BH, AE, JJT, and CL wrote the manuscript. All authors have seen and approved the manuscript and its contents and are aware of the responsibilities connected to authorship.

## Electronic supplementary material

Below is the link to the electronic supplementary material.

Supplementary file1 (PDF 1792 kb)

Supplementary file2 (PDF 195 kb)

Supplementary file3 (PDF 239 kb)

Supplementary file4 (PDF 215 kb)

Supplementary file5 (PDF 144 kb)

Supplementary file6 (PDF 188 kb)

Supplementary file7 (PDF 55 kb)

## Abbreviations

IBA: Indole-3-butyric acid
OPDA: Proto-methyl-jasmonic acid
PTD: Peroxisomal targeting domain
PTS1: Peroxisomal targeting signals type 1
SnRK1: SNF1-related protein kinase 1
T6P: Trehalose-6-phosphate
TPP: Trehalose-6-phosphate phosphatase
TPS: Trehalose-6-phosphate synthase
