# Supplementary material for: Multi-targeted trehalose-6-phosphate phosphatase I harbors a novel peroxisomal targeting signal 1 and is essential for flowering and development
Source: Planta. 2020 Apr 18;251(5):98. doi: 10.1007/s00425-020-03389-z (PMC7214503; doi:10.1007/s00425-020-03389-z)
Supplement: Supplementary file 1 — Supplementary file1 (PDF 1792 kb) [file 425_2020_3389_MOESM1_ESM.pdf]

Supplementals for

Multi-targeted trehalose-6-phosphate phosphatase I harbors a novel peroxisomal targeting signal 1 and is essential for flowering and development

Amr R.A. Kataya[1,2*], Ahmed Elshobaky[1,3], Behzad Heidari[1,4], Nemie-Feyissa Dugassa[1], Jay J. Thelen[2], Cathrine Lillo[1]

[1]University of Stavanger, Centre for Organelle Research, Faculty of Science and Technology, N-4036 Stavanger, Norway.

[2]Present Address: Christopher S. Bond Life Sciences Center, Department of Biochemistry, University of Missouri, Columbia, MO, United States

[3]Botany Department, Faculty of Science, Mansoura University, 35516 Mansoura, Egypt

[4]Department of Plant Biology, Scholl of Biology, College of Science, University of Tehran, Tehran, Iran

Corresponding author: Amr R.A. Kataya; amr.kataya@missouri.edu, dramrkataya@gmail.com, T. +1 573-452-3412, Fax:+1 573-884-9395, https://orcid.org/0000-0003-1349-0363

```
At-NP196572_2          1 ---------------------------------------------MSASQNIVVSETTMSIIPNNNNN----NNNSSSQK
Cr-XP006287992_1       1 ----------------------------------------------MSASQNVLVSETTMSSIIPNNNN--------SSS
Es-XP006399500_1       1 ----------------------------------------------MSASQNVVVSETTRSSIITSSNNN----S--SSS
Rc-XP002533145_1       1 -----------------------------------------------MTNQNVVVADAKSTIN-LATTVHVSNSSIFTTA
Tc-EOX92150_1          1 -----------------------------------MVSFFEERPWNMGAGQNVISDAKSLNMSITM---------TVSAQ
Pv-ESW35633_1          1 ----------------------------------------------MTQQNVVVSDTKSVP----------LTVFPTPAQ
Pp-EMJ06566_1          1 ----------------------------------------------MTKQNVVVSDAEAIMNMAISVAVSNSSIFTAVAE
Pt-XP002307096_1       1 -----------------------------------------------MTNQNVVVADTNSGIN-LAITVHVTNSSIFTTA
Gm-XP006601938_1       1 -----------------------------------------------MTQQNVVVSDTKSGVS----------LTVFPTT
Ca-XP004504061_1       1 -----------------------------------------------MTQQNVVVSETKSGIN------------AAITM
Ca-XP004502438_1       1 -----------------------------------------------MTQQNVLVSDTKSGV------------TVFPTT
Pv-ESW31633_1          1 ----------------------------------------------MTQQNVVLSKTKSIN------------RDLTVAQ
Gm-XP003532832_1       1 ------------------------------------------------MTQNAIVSKTKSGIK------------GDIAV
Gm-XP003524269_1       1 ------------------------------------------------MTQNAIVSKTKSGIN------------RDITV
Cs-XP006464217_1       1 -----------------------------------MVSFNIEAERENMGNQNVLIVDAFNLTLSTEGLPNPS-IYSIASQ
Cc-XP006428166_1       1 -----------------------------------MVSFNIEAERENMGNQNVLIVDAFNLTLSTEGLPNPS-IYSIASQ
Cc-XP006428167_1       1 MGKFHFRISQSGFIRKKHFLSTSGKLKSCFFTSSSIVSFNIEAERENMGNQNVLIVDAFNLTLSTEGLPNPS-IYSIASQ
Rc-XP002510945_1       1 --------------------------------------------MDLKSNHNAPVLTDPAPISKSRLG-----VHSSLLP
Vv-XP002265679_2       1 --------------------------------------------MDLKSNHSSPVLTDPPPVNKSRF-------------
Vv-CAN71555_1          1 -------------------------------------------MDLKSNHSSPVLTDPPVNKSRFG-----IRSNLLPY-
Tc-EOY22902_1          1 -------------------------------------------MDLKSNHTAPIIADPAISKSRLG-----VHSSLLAY-
Pp-EMJ19320_1          1 -------------------------------------------MDLKSNHAAPVLTDPTLSKSRLG-----VPSSLLQY-
Pt-XP002318886_1       1 --------------------------------------------MDLKSNHNAPVLTDSAPLSKSRLRGY---HHGLMLP
Fvs_v-XP004307816_1    1 _1 --------------------------------------------MDLKSNHISPVLTDPA_1 PLGKSRLG-----V
Cc-XP006421675_1       1 --------------------------------------------MDLKTNHSAPVLTDPAPISKSRLG-----IHSSLLP
Cs-XP006490109_1       1 --------------------------------------------MDLKTNHSAPVLTDPAPISKSRLG-----VHSSLLP
Cs-XP004148142_1       1 --------------------------------------------MDLKSNHNSPILTDPAPISKSRLG-----VHLNMLP
Cs-XP010453031_1       1 -----------------------------------MVSFFVEKPQTMSASQNVFVSETTMSSIIPNNNNN-------SSS
Cs-XP010453030_1       1 --------------------------------------------------------------------------------
Th-XP010519807_1       1 -------------------------------------MVSFVEENQRMATQNVVVSESKSGIIAMAVSNS----AVFTPS
Th-XP010547623_1       1 -------------------------------------MVSFVEKRERMASQNVVASEAKSGIITVTV-------AVANPS
Th-XP010519815_1       1 -----------------------------------------------MATQNVVVSESKSGIIAMAVSNS----AVFTPS
Th-XP010547624_1       1 -----------------------------------------------MASQNVVASEAKSGIITVTV-------AVANPS
Jc-XP012093071_1       1 -----------------------------------------------MTNQNVVVSDTKSTIN-LAITVHVSNSNIFTTA
Gr-KJB18196_1          1 ---------------------------MLGLYLFIAVSFIEERPKNIAAGQNMVSDPK-------------------SAQ
Ga-KHG13010_1          1 ----------------------------------------------------MVSDPK-------------------SAQ
Pv-XP007163639_1       1 -----------------------------------------------MTQQNVVVSDTKSGVP----------LTVFPTP
Mt-XP013446829_1       1 -----------------------------------------------MTQKNVVVSETKTGIN----------GSGTITV
Pp-XP007205367_1       1 -----------------------------------------------MTKQNVVVSDAEAEIMNMAISVAVSNSSIFTAV
Gr-KJB49173_1          1 -----------------------------------MVSFFEDSAKNIAGKNVMFTDAKSLNMSITV---------TVSAQ
Gr-XP012437482_1       1 -----------------------------------------------------MFTDAKSVLNMSITV---------TVS
Va-KOM30696_1          1 ----------------------------------------------MTQQNVVLSKTKSIN------------RDLTVAQ
Ca-XP004504060_1       1 -----------------------------------------------MTQQNVVVSETKSGIN------------AAITM
Pe-XP011030992_1       1 -----------------------------------------------MTNQNVVVADTSSGIN-LAITVHVTNSSIFTTA
Mas_m-XP009380850_1    1 _1 -----------------------------------------------MTKQNVVPAEAVA_1 AITAAAA-------
Cs-KDO56609_1          1 --------------------------------------------------------------------------------
Ga-KHG18855_1          1 ----------------------------------------------------MITDAKSLNMSITV---------TVSTQ
Pt-XP002307388_2       1 ------------------------------------------------------MALSKSLFS--------------PVV


At-NP196572_2         32 ---LPP---CL-ISISKKKLL--KNIDIINGG-GQRINWVDSMRASSPTHLKSLPSS------ISTQQQLNSWIMQHPSA
Cr-XP006287992_1      27 QK---LPP---GL-IPVSKKQLL--KNIDIINGGAGQRINAWVDSMRASSPTHLRSLPSS------ISSQQQLNSWIMQH
Es-XP006399500_1      29 PKPSTAPP---GL-ISISKKKLL--KNLDVINGG--QRINAWVDSMRASSPTHLKSLPSS------IYSQQQLNSWIMHH
Rc-XP002533145_1      33 VQKPPAAP---GGYISISRKKLL--KNLE-INGG--ARINAWVDSMRASSPTHLKSTP---------SLTEDQGSWTLHH
Tc-EOX92150_1         37 KPPAPP---GY-ISISRKKLL--QNLE-INAG--GRINWVDSMRASSPTHIKSTPS---------LIDDQSSWNLQHPSA
Pv-ESW35633_1         25 KKPPAAP--GGYISISRRRVL--KNLE-INGD--QRINWVESMRASSPTHLKSTP---------SFSQEQNSWILHHPSA
Pp-EMJ06566_1         35 KPPVGP---PGYISISRKRFLNLKNLDSINGA--ERINWVDSMRASSPTHLKSSS---------FLKEDQSSWILQHPSA
Pt-XP002307096_1      33 AQKPPAAP---GGYISISRKKLL--KNLE-ISGG--ARFNAWVNSMRTSSPTHVKSTP---------SANDDQSSWILHH
Gm-XP006601938_1      24 TQK-PPAAP--GGYISIPRRRVL--KNLE-INGG--QRINAWVESMRASSPTHHKSTP----------LSQEHNSWILHH
Ca-XP004504061_1      22 TQKPPAPP---AGYIPIPRRRIL--KNLE-INAG--QRINTCIDSMRASSPTHVKSTP---------SFAEEYNSWILRH
Ca-XP004502438_1      22 SQKPPPAPG--GGYISIQRRWVL--KDLE-INGG--QRINAWLESMRASSPTHLKSTP---------SFAEEQTSWILHH
Pv-ESW31633_1         23 KPPAAP---GGYIPIPRRRVL--KNLE-INGG--QRINWVDSMRASSPTQAKSTS---------SFAEEHSSWILRHPSA
Gm-XP003532832_1      21 AQKPLAAPPPPG-YIPIPRRRVL--KNLE-INGG--QRINAWVDSMRASSPTNSKSTS---------SLAEEHSSWILRH
Gm-XP003524269_1      21 PQKPLAAAAAAGGYIPIPRRRVL--KNLE-IN--------AWVDSMRSSSPTNSKSTS---------SLAEEHSTWILRH
Cs-XP006464217_1      45 QKRPPVVP---CGGYTNKKKLML--QNLETNAAAGARPNNCWVESIRASSPTHMKSITP--------SLLEEKRAWIIRH
Cc-XP006428166_1      45 QKRPPVVP---CGGYTNKKKLML--QNLETNAAAGARPNNCWVESIRASSPTHMKSITP--------SLLEEKRAWIIRH
Cc-XP006428167_1      80 QKRPPVVP---CGGYTNKKKLML--QNLETNAAAGARPNNCWVESIRASSPTHMKSITP--------SLLEEKRAWIIRH
Rc-XP002510945_1      32 Y-SSAGGAFSSNLWLTIPRKKTG---VLDDVRSN------NWLDAMKSSSPPHKRITKDLSSEFSSVDVDFAYRTWTLKY
Vv-XP002265679_2      24 -----GAAFSSTLFLTIPRKKNG---VLDDVRSS------SWLDAMKSSSPTHKKITKDFNTVLASTDTDVTYRTWLLKY
Vv-CAN71555_1         32 SPPGAAFSSTLFLTIPRKKNG---VLDDVRSS------WLDAMKSSSPTHKKITKDFNTVLASTDTDVTYRTWLLKYPSA
Tc-EOY22902_1         32 SPPGAVFSPNLFLTVPRKKTG---ILDDVRAS------WLDAMKSSSPPHK-KTREFNNELASADTDVAYRTWMVKYPSA
Pp-EMJ19320_1         32 SPPGAAFSAGLILAIPRRKAG---LLDDVRSN------WLDAMKSSSPPHRKITKDVNNEPVANEADIAYHTWMVKYPSA
Pt-XP002318886_1      34 Y-SPSGAPFSSNLLLSIPRRKTG---VLDDVRSC------GWLDAMKSSSPTHKKFAKDINHELSAPDPEVAYRTWLLKY
Fvs_v-XP004307816_1   26 HSSLLSY-SPPGTAFPADLLLTIPRRKNA---FLDDVRSS------_1 SWLDAMKSSSPPHRKITKDVNNDSAATEYDI
Cc-XP006421675_1      32 Y-SPSG-TFPSDLFLAIPRKKTG---VLDDVRAC------SWLDAMKSSSPPPKWMAKESNNEFLSTDTDVAYRTWQRQY
Cs-XP006490109_1      32 Y-SPSG-TFPSDLFLAIPRKKTG---VLDDVRAR------SWLDAMKSSSPPPKWMAKESNNEFSSTDTDVAYRTWQRQY
Cs-XP004148142_1      32 YVSAPGVGFSPNMLL-IPRKKTG---VLDDVRAS------SWLDAMKSSSPPPNRISKDDMNELPSLDPDILYRNWMIKY
Cs-XP010453031_1      39 QK---LPP---GL-ISISKKNLL--KNIDIINGGSGQRINAWVDSMRASSPTHLKSLPSS------ISSQHQLNSWIMQH
Cs-XP010453030_1       1 ---------------------------------------------MRASSPTHLKSLPSS------ISSQHQLNSWIMQH
Th-XP010519807_1      40 AQKPPTAP---GY-ISISRKKLL--KNLD-INGG--HRINAWVDSMRASSPTHLKSVPS--------LSADERNSWIMQH
Th-XP010547623_1      37 AQKPPTAP---GY-ISISRKKLL--HNLE-INGG--HRINAWVDSMRASSPTHLKSVPS--------LSPEERNSWIIQH
Th-XP010519815_1      30 AQKPPTAP---GY-ISISRKKLL--KNLD-INGG--HRINAWVDSMRASSPTHLKSVPS--------LSADERNSWIMQH
Th-XP010547624_1      27 AQKPPTAP---GY-ISISRKKLL--HNLE-INGG--HRINAWVDSMRASSPTHLKSVPS--------LSPEERNSWIIQH
Jc-XP012093071_1      33 AQKPPAAP---AGYISISRKKLL--KNLE-INGG--PRINAWVESMRASSPTHLKSTPP--------SLTDDQRSWILHH
Gr-KJB18196_1         35 KPPAPP---GF-ISISRKKLL--QNLE-INAG--ARVNWVDSMRASSPTHMKSAPS---------IADDQGSWNLNHPSA
Ga-KHG13010_1         10 KPPAPP---GF-ISISRKKLL--QNLE-INAG--ARVNWVDSMRASSPTHMKSAPS---------TADDQGSWNLNHPSA
Pv-XP007163639_1      24 AQKKPPAAP--GGYISISRRRVL--KNLE-INGD--QRINAWVESMRASSPTHLKSTP---------SFSQEQNSWILHH
Mt-XP013446829_1      24 AQKPPAAP---GGYVHIPRRRIL--KNLE-INGG--QRINTWIDSMRASSPTHVKSSP---------SLAEEYNSWILRH
Pp-XP007205367_1      34 AEKPPVGP---PGYISISRKRFLNLKNLDSINGA--ERINSWVDSMRASSPTHLKSSS---------FLKEDQSSWILQH
Gr-KJB49173_1         37 NPLASP---GYKISIKTKKLL--QNRE-IISG--ARVNWVDSMRASSPTHTKSTSS---------VTDDQVTWNLHHPSA
Gr-XP012437482_1      19 AQNPLASP---GYKISIKTKKLL--QNRE-IISG--ARVNAWVDSMRASSPTHTKSTSS---------VTDDQVTWNLHH
Va-KOM30696_1         23 KPPAAP---GGYIPIPRRRVL--KNLE-INGG--QRINWVDSMRASSPTHAKSSS---------SLAEEHSSWILRHPSA
Ca-XP004504060_1      22 TQKPPAPP---AGYIPIPRRRIL--KNLE-INAG--QRINTCIDSMRASSPTHVKSTP---------SFAEEYNSWILRH
Pe-XP011030992_1      33 KQKPPAAP---GGYITISRNKLL--KNLE-ISGG--ARFNAWVNSMRTSSPTHVNSTP---------SVNDDQSSWILHH
Mas_m-XP009380850_1   21 -VASSSPLYPYPPP------RSFQKKYLS---QLD-FAGG---RIG_1 TWVESMKASSPTHTKAVGAIG-----VPVDE
Cs-KDO56609_1          1 -------------------ML--QNLETNAAAGARPNNWVESIRASSPTHMKSITP--------SLLEEKRAWIIRHPSA
Ga-KHG18855_1         20 NPLASP---GHNISIKTKKLL--QNHE-ISAG--ARVNWVDSMRASSPTHTKSTSS---------VTDDQVTWNLHHPSA
Pt-XP002307388_2      13 PKPLPAAP---GGYFTISRKMFA--KKTE--TGG---KTNSWADSMRDSSPTRVKSTTS-------LSEIEEKNTWIVNH
```

```
At-NP196572_2          96 LEKFEQIMEASRGKQIVFLDYDGTLSPIVDDPDKAFMSSKMRRTVKKLAKCFPTAIVTGRCIDKVYNFVKLAELYYGSHG
Cr-XP006287992_1       92 PSALEMFERIMEASGGKQIVMFLDYDGTLSPIVDDPDKAYMSSKMRRTVKKLAKCFPTAIVTGRCIDKVYNFVKLAELYY
Es-XP006399500_1       95 PSALEMFEQIMEASGGKQIVMFLDYDGTLSPIVDDPDKAFMSSKMRRTVKKLAKCFPTAIVTGRCIDKVYNFVKLAELYY
Rc-XP002533145_1       96 PSALDMFEQIIDASKGKQIVMFLDYDGTLSPIVDDPDRAFMSKKMRRATVRKLAKCFPTAIVSGRCRDKVYNFVRLAELYY
Tc-EOX92150_1          99 LEMFEQIIDASTGKQIVFLDYDGTLSPIVEDPDRAFMSKKMRKTVRKLAKCFPTAIVSGRCRDKVYKFVKLAELYYGSHG
Pv-ESW35633_1          89 LDMFDQIIDASKGKQIVFLDYDGTLSPIVEDPDRAFMSHSMRKTVRKVARCFPTAIVTGRCKDKVYNFVQLAELYYGSHG
Pp-EMJ06566_1         101 LDMFEQIIDASKGKQIVFMDYDGTLSPIVEDPDRAFMSDAMRKTVKKVARCFPTAIVSGRCRDKVYKFVKLAELYYGSHG
Pt-XP002307096_1       96 PSALEMFEQIIDASKGKQIVMFLDYDGTLSPIVDDPDRAFMSKKMRRATVRKLARFFPTAIVSGRCRDKVYNFVRLAELYY
Gm-XP006601938_1       86 PSALDMFDQIIDASKGKQIVMFLDYDGTLSPIVDDPDRAFMSDSMRKTVRKLARCFPTAIVTGRCKDKVYNFVRLAELYY
Ca-XP004504061_1       85 PSALDMFEQIKDASKGKQIVMFLDYDGTLSPIVDDPDRAFMSESMRKTVRKLARCFPTAIVTGRCIDKVYNFIRLAELYY
Ca-XP004502438_1       86 PSALDMFDQIIEASKGKQIVMFLDYDGTLSPIVEDPDRAFMSDSMRKTVRKLARYFPTAIVTGRCKDKVYNFVRLAELYY
Pv-ESW31633_1          86 LDMFEQIMDASRGKQIVFLDYDGTLSPIVDDPDRAFMSDSMRKTVRKLARCFPTAIVTGRCKDKVYNFVRLAELYYGSHG
Gm-XP003532832_1       86 PSALDMFEQIMDASRGKQIVMFLDYDGTLSPIVDDPDHAFMSDSMRRTVRKLARCFPTAIVTGRCKDKVYSFVRLAELYY
Gm-XP003524269_1       81 PSALDMFEQIMDASRGKQIVMFLDYDGTLSPIVDDPDRAFMSDSMRKTVRKLARCFPTAIVTGRCKDKVYNFVRLAELYY
Cs-XP006464217_1      112 PSALDMFHEITEASKGKQIVMFLDYDGTLSPIVENPDRAFMSGKMRRAVRQLAKYFPTAIVTGRCRDKVYDFVKLAELYY
Cc-XP006428166_1      112 PSALDMFHEITEASKGKQIVMFLDYDGTLSPIVENPDRAFMSGKMRRAVRQLAKYFPTAIVTGRCRDKVYDFVKLAELYY
Cc-XP006428167_1      147 PSALDMFHEITEASKGKQIVMFLDYDGTLSPIVENPDRAFMSGKMRRAVRQLAKYFPTAIVTGRCRDKVYDFVKLAELYY
Rc-XP002510945_1      102 PSAIASFEQITNFAKGKRIALFLDYDGTLSPIVDNPDCAFMSNAMRTAVKSVAKCFPTAIISGRSRDKVYEFVGLTELYY
Vv-XP002265679_2       90 PSALKSFEQITNYAKGKRIALFLDYDGTLSPIVDNPDRAFMSDCMRATVRNVAKFFPTAIISGRSRDKVYNFVGLTELYY
Vv-CAN71555_1         103 LKSFEQITNYAKGKRIAFLDYDGTLSPIVDNPDRAFMSDCMRATVRNVAKFFPTAIISGRSRDKVYNFVGLTELYYGSHG
Tc-EOY22902_1         102 LTSFEQITNFAKGKRMAFLDYDGTLSPIVDNPDCAFMSTDMRAAVEKVAKYFPTAIISGRSRDKVYEFVGLTDLYYGSHG
Pp-EMJ19320_1         103 LTYFEQITNYAKGKRIAFLDYDGTLSPIVDNPDCAFMSDAMRAAVRVAKHFPTAIISGRSHDKVYEFVGLKELYYGSHG
Pt-XP002318886_1      104 PSAIASFEQIANFAKGKRIALFLDYDGTLSPIVENPDNAFMSADMRSIVKEVAKYFPTAIISGRSRDKVYEFVGLTELYY
Fvs_v-XP004307816_1    93 AYRTWMVKYPSALTSFEQISNYAKGKRIA_1 LFLDYDGTLSPIVDNPDCAFMSDAMRMAVKRVAKHFPTAIISGRSHDK
Cc-XP006421675_1      101 PSALTSFEQILKSAKGKRIALFLDYDGTLSPIVDNPDCAFMSDAMRAVVKNVAKYFPTAIISGRSRDKVYEFVGLAELYY
Cs-XP006490109_1      101 PSALTSFEQIVKSAKGKRIALFLDYDGTLSPIVDNPDCAFMSDAMRAVVKNVAKYFPTAIISGRSRDKVYEFVGLAELYY
Cs-XP004148142_1      102 PSAIASFDQIVPHAKGKRIALFLDYDGTLSPIVDNPDGAFMSDAMRATVKEAAKYFPTAIISGRSRDKVYEFIGLKELYY
Cs-XP010453031_1      104 PSALEMFDRIIEASGGKQIVMFLDYDGTLSPIVDDPDKAFMSSKMRRTVKKLAKCFPTAIVTGRCIDKLYNFVKLAELYY
Cs-XP010453030_1       30 PSALEMFDRIIEASGGKQIVMFLDYDGTLSPIVDDPDKAFMSSKMRRTVKKLAKCFPTAIVTGRCIDKLYNFVKLAELYY
Th-XP010519807_1      103 PSALDMFEQIIEASRGKQIVMFLDYDGTLSPIVEDPDRAFMSSKMRRTVRKVAKCFPTAIVSGRCRDKVYSFVKLAELYY
Th-XP010547623_1      100 PSALDMFEQIIEASRGKQIVMFLDYDGTLSPIVDDPERAFMSSKMRRTVRKLAKCFPTAIVSGRCRDKVYNFVKLAELYY
Th-XP010519815_1       93 PSALDMFEQIIEASRGKQIVMFLDYDGTLSPIVEDPDRAFMSSKMRRTVRKVAKCFPTAIVSGRCRDKVYSFVKLAELYY
Th-XP010547624_1       90 PSALDMFEQIIEASRGKQIVMFLDYDGTLSPIVDDPERAFMSSKMRRTVRKLAKCFPTAIVSGRCRDKVYNFVKLAELYY
Jc-XP012093071_1       97 PSALDMFEQIIDASKGKQIVMFLDYDGTLSPIVDDPDRAFMSKKMRRATVRRLARCFPTAIVSGRCRDKVYNFVRLAELYY
Gr-KJB18196_1          97 LDMFEQIIDASKGKQIVFLDYDGTLSPIVADPDRAFMSKKMRKTVRKLAKCFPTAIVSGRCRDKVYNFVKLAELYYGSHG
Ga-KHG13010_1          72 LDMFEQIIDASKGKQIVFLDYDGTLSPIVADPDRAFMSKKMRKTVRKLAKCFPTAIVSGRCRDKVYNFVKLAELYYGSHG
Pv-XP007163639_1       88 PSALDMFDQIIDASKGKQIVMFLDYDGTLSPIVEDPDRAFMSHSMRKTVRKLARCFPTAIVTGRCKDKVYNFVQLAELYY
Mt-XP013446829_1       87 PSALDMFEQIMDAAKGKQIVMFLDYDGTLSPIVDDPDRAFMSESMRKTVKKLARCFPTAIVTGRCIDKVYNFVRLDELYY
Pp-XP007205367_1      100 PSALDMFEQIIDASKGKQIVMFMDYDGTLSPIVEDPDRAFMSDAMRKTVKKVARCFPTAIVSGRCRDKVYKFVKLAELYY
Gr-KJB49173_1         100 LEMFEQIIDASKGKQIVFLDYDGTLSPIVEDPDRAFMSKKMRKTVRKLAKCFPTAIVSGRCRDKVYKFVKLAELYYGSHG
Gr-XP012437482_1       82 PSALEMFEQIIDASKGKQIVMFLDYDGTLSPIVEDPDRAFMSKKMRKTVRKLAKCFPTAIVSGRCRDKVYKFVKLAELYY
Va-KOM30696_1          86 LDMFEQIMDASRGKQIVFLDYDGTLSPIVDDPDRAFMSDSMRKTVRKLARCFPTAIVTGRCKDKVYNFVRLAELYYGSHG
Ca-XP004504060_1       85 PSALDMFEQIKDASKGKQIVMFLDYDGTLSPIVDDPDRAFMSESMRKTVRKLARCFPTAIVTGRCIDKVYNFIRLAELYY
Pe-XP011030992_1       96 PSALEMFGQIIDASKGKQIVMFLDYDGTLSPIVEDPDRAFMSKQMRRATVRKLARFFPTAIVSGRCRDKVYNFVRLAELYY
Mas_m-XP009380850_1    79 QSAAWIMRHPSALTKFEQIMSASKGKQIV_1 MFLDYDGTLSPIVEDPDSAFMTDAMRAAVRNVARYFPTAIVTGRCLEK
Cs-KDO56609_1          52 LDMFHEITEASKGKQIVFLDYDGTLSPIVENPDRAFMSGKMRRAVRQLAKYFPTAIVTGRCRDKVYDFVKLAELYYGSHG
Ga-KHG18855_1          83 LEMFEQIIDASKGKQIVFLDYDGTLSPIVEDPDRAFMSKKMRNTVRKLAKCFPTAIVSGRCRDKVYKFVKLAELYYGSHG
Pt-XP002307388_2       76 PSALNMFEQIVNGSKGKQIVMFLDYDGTLSPIVEDPDRAFMTNEMREAVRDVARYFPTAIVTGRCRDKVYSFVRLAGLYY
```

```
At-NP196572_2         176 MDIKGPAKGFSRHKR-----------VKQSLLYQPANDYLPMIDEVYRQLLEKTKTPGAKVENHKFCASVHFRCVDEKKW
Cr-XP006287992_1      172 AGSHGMDIKGPAKGFPRHKR-----------VKQSLLYQPASDYLPMIDEVYRKLLEKTKSTPGARVENHKFCASVHFRC
Es-XP006399500_1      175 AGSHGMDIKGPSKGVSRQKR-----------LKQSLLYQPASDYLPMIDEVYRKLLETTKSTPGAKVENHKFCASVHFRC
Rc-XP002533145_1      176 AGSHGMDIKGPAKG-SKYKKG-----------SEALIFQPASEFLPMIDEVYKELVEKTKSTPGAFVENNKFCVSVHYRC
Tc-EOX92150_1         179 MDIKGPEKR-SKSNK-----------DSESVLFQPASEFLPMIDEVYKQLVETTKTPGAKVENNKFCLSVHFRCVDEKKW
Pv-ESW35633_1         169 MDIKGPTRS-SKYNKDA---------KAEAILFQPASDFLPMIDEVYQQLVEKTKTPGAMVENNKFCLSVHFRCVDEKKW
Pp-EMJ06566_1         181 MDIKGPAKG-SKYKTG-----------SQGVLCQPASEFLPMIDEVYKLLVEKTKTPGAKVENNKFCLSVHFRCVDEKKW
Pt-XP002307096_1      176 AGSHGMDIKGPAKG-SKYKKG-----------SEGVVFQAGSEFLPMIDEVYKELVEKTKTTPGAKVENNKFCLSVHFRC
Gm-XP006601938_1      166 AGSHGMDIKGPTRS-SKYNKDS---------KTEAILCQPASDFLPLIDEVYQQLVEKTKSTPGALVENNKFCLSVHFRC
Ca-XP004504061_1      165 AGSHGMDIKGPTKE-SKYNK-----------VTEALLFQPAREFVPMINEVYQKLVEKTKSTPGVIVENHKFCASVHFRC
Ca-XP004502438_1      166 AGSHGMDIKGPTRS-SKYNKGS---------KAQAVLYQPASEFLPMIDEVYKQLVDKTKSTPGAKVENNKFCVSVHFRC
Pv-ESW31633_1         166 MDIQGPTRD-SKYSNKD---------KGEPLLFQPASEFLPMIDEVYNQLVEKMKIPGARVENNKFCASVHFRCVDEKKW
Gm-XP003532832_1      166 AGSHGMDIQGPTRD-CKYS-KD---------KGEPVLFQPASEFLPMIDEVHQLVEKMKSIPGAKVENNKFCCSVHFRC
Gm-XP003524269_1      161 AGSHGMDIQGPTRT-SKYSNKD---------KGEPVLFQPASEFLPMIDEVYHQLVEKMKSIPGAMVENNKFCCSVHFRC
Cs-XP006464217_1      192 AGSHGMDIKGPTKG-LKYNQ-----------KSKVVNFQPASEFLPLIDKVYKVLVEKTKSTPGARVENNKFCISVHFRC
Cc-XP006428166_1      192 AGSHGMDIKGPTKG-LKYNQ-----------KSKVVNFQPASEFLPLIDKVYKVLVEKTKSTPGARVENNKFCISVHFRC
Cc-XP006428167_1      227 AGSHGMDIKGPTKG-LKYNQ-----------KSKVVNFQPASEFLPLIDKVYKVLVEKTKSTPGARVENNKFCISVHFRC
Rc-XP002510945_1      182 AGSHGMDIMGPVRRQSVPDEHSNCIRSADKQGKEVNLFQPASEFLPMIDEVYSSLVASTNHIKGAKVENNKFCVSVHYRN
Vv-XP002265679_2      170 AGSHGMDIMGPVR-HSISNDHPNCIRLTDKQGKEVNLFQPASEFLPMIDEVFRSLVRCTKEIKGAEVENNKFCVSVHYRN
Vv-CAN71555_1         183 MDIMGPVR-HSISNDHPNCIRLTDKQGKEVNLFQPASEFLPMIDEVFRSLVRCTKEIKGAEVENNKFCVSVHYRNVDEKYW
Tc-EOY22902_1         182 MDIMGPVR--QFSDDHANCIRSTDKQGKEVNLFQPASEFLPMIDEVFNSLVNSTKEIKGAEVENNKFCVSVHYRNVDEKNW
Pp-EMJ19320_1         183 MDIMGPGR-QSTADDHRNGFRTSDKQGKDVNLFQPAAEFLPMIGEVYESLVESTKEIEGAKVENNKFCVSVHYRNVDEKSW
Pt-XP002318886_1      184 AGSHGMDIMGPVR-QSVSDDHRNCIKSTDKQGNEVNLFQPAREFLPMIDEVYSSLVRITEDIKGATVENNKFCVSVHYRN
Fvs_v-XP004307816_1   170 VYEFVGLNELYY_1 AGSHGMDIMAPVL-QSVVGDHRHGFRSTDKQGNDVNVFQPAAEFLPMIGEVLKSLEESTK_1 DI
Cc-XP006421675_1      181 AGSHGMDIMGPVR-QSIPNDNADTIQSTGKQGKEVNLFQPAREFLPMIDAVFHSLMENTKEINGVKVENNKFCVSVHYRN
Cs-XP006490109_1      181 AGSHGMDIMGPVR-QSIPNDNADTIQSTDKQGKEVNLFQPAREFLHMIDAVFHSLMENTKEINGVKVENNKFCVSVHYRN
Cs-XP004148142_1      182 AGSHGMDIMVSDR-----------HSADNQGKEV-MFQPASEFLPLIDEVYKSLIEITKGIAGAKVENNKFCVSVHYRN
Cs-XP010453031_1      184 AGSHGMDIIGPAKGFSRHKR-----------VKQSLLYQPANDYLPMIDEVHKQLLEKTKSTPGVKVENHKFCASVHFRC
Cs-XP010453030_1      110 AGSHGMDIIGPAKGFSRHKR-----------VKQSLLYQPANDYLPMIDEVHKQLLEKTKSTPGVKVENHKFCASVHFRC
Th-XP010519807_1      183 AGSHGMDIKGPTKGFSKYKK-----------DKQSVLFQPASEFLPLIDEVYKQLVENTKSVPGAKVENNRFCVSVHFRC
Th-XP010547623_1      180 AGSHGMDIKGPTKGSSKYQK-----------DNPSVVFQPASEFLPIIDEVYKELVDKTKSTPGAMVENNKFCLSVHFRC
Th-XP010519815_1      173 AGSHGMDIKGPTKGFSKYKK-----------DKQSVLFQPASEFLPLIDEVYKQLVENTKSVPGAKVENNRFCVSVHFRC
Th-XP010547624_1      170 AGSHGMDIKGPTKGSSKYQK-----------DNPSVVFQPASEFLPIIDEVYKELVDKTKSTPGAMVENNKFCLSVHFRC
Jc-XP012093071_1      177 AGSHGMDIKGPAKG-SKYKKG-----------NEGLIFQPASEFLPMIDEVYKELVEKTKSTAGAKVENNKFCVSVHFRC
Gr-KJB18196_1         177 MDIKGPEKG-SKSNK-----------DTESVLFQPASEFLPMIDEVYKQLVETTKTPGAKVENNKFCLSVHFRCVDEKKW
Ga-KHG13010_1         152 MDIKGPEKC-SKSNK-----------DTESVLFQPASEFLPMIDEVYTQLVETTKTPGAKVENNKFCLSVHFRCVDEKKW
Pv-XP007163639_1      168 AGSHGMDIKGPTRS-SKYNKDA---------KAEAILFQPASDFLPMIDEVYQQLVEKTKSTPGAMVENNKFCLSVHFRC
Mt-XP013446829_1      167 AGSHGMDIKGPTKE-SKYNKNN---------KAEEVLFQPAREFVPMINEVYEQLVEKTKSTPGARVENHKFCTSVHFRC
Pp-XP007205367_1      180 AGSHGMDIKGPAKG-SKYKTG-----------SQGVLCQPASEFLPMIDEVYKLLVEKTKSTPGAKVENNKFCLSVHFRC
Gr-KJB49173_1         180 LDIKGPEKR-SKSKS-----------DGESVLFQPASEFLPMIDEVYKQLVDTTKTPGAKVENNKFCLSVHFRCVDEKKW
Gr-XP012437482_1      162 AGSHGLDIKGPEKR-SKSKS-----------DGESVLFQPASEFLPMIDEVYKQLVDTTKSTPGAKVENNKFCLSVHFRC
Va-KOM30696_1         166 MDIQGPTRD-SKYSNKD---------KGEPVLFQPASEFLPMIDEVYNQLVEKMKIPGARVENNKFCASVHFRCVDEKKW
Ca-XP004504060_1      165 AGSHGMDIKGPTKE-SKYNKNN---------KAEALLFQPAREFVPMINEVYQKLVEKTKSTPGVIVENHKFCASVHFRC
Pe-XP011030992_1      176 AGSHGMDIKGPAKG-SKYKKG-----------SEGVVFQAGSEFLPMIDEVYKELVERTKTTPGAKVENNKFCLSVHYRC
Mas_m-XP009380850_1   156 VIDFVGLTELYY_1 AGSHGMDIKGPAKP--RHTKA----------KAKSVLFQPAREFLPMIDGVYRSLLERTK_1 ST
Cs-KDO56609_1         132 MDIKGPTKG-LKYNQ-----------KSKVVNFQPASEFLPLIDKVYKVLVEKTKTPGARVENNKFCISVHFRCVDEKKW
Ga-KHG18855_1         163 LDIKGPEKR-SKSKNERVCSLVKV-MDGESVLFQPASEFLPMIDEVYKQLVDTTKTPGAKVENNKFCLSVHFRCVDEKKW
Pt-XP002307388_2      156 AGSHGMDIKGPSKNCCINKK-----------DYQGVLFQPASDFLPMIDEVYNALLERTKYIPGARVEDNKFCISVHFRC
```

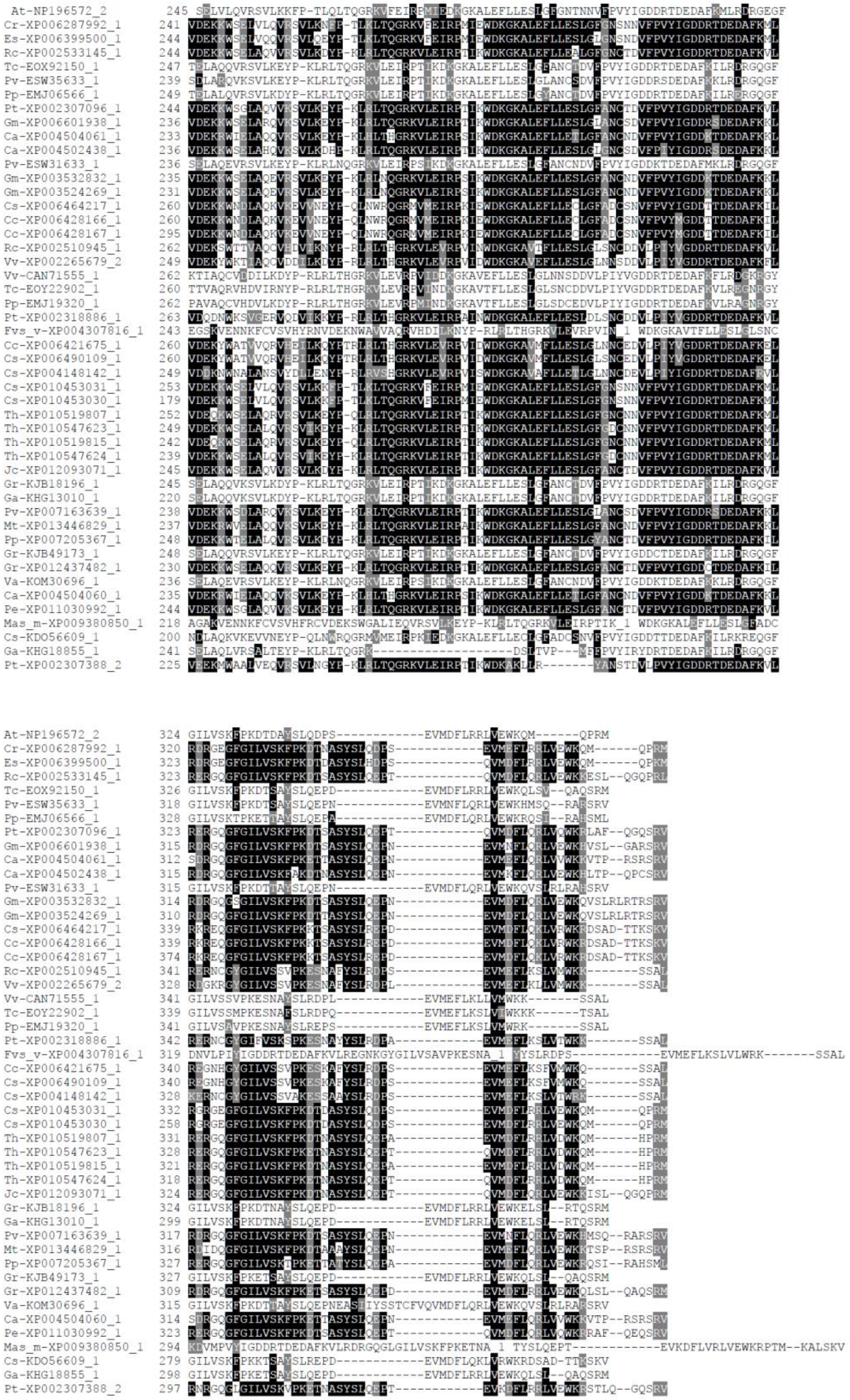

Supplementary Fig. S1 TPPI homolog sequences alignment. Sequences of full-length TPPI homologs that harbors a conserved PTS1 were identified by BLAST (see Fig. 1), aligned by ClustalW/MEGA6 (Tamura et al. 2013), and conserved residues were shaded by BoxShade (http://www.ch.embnet.org/software/BOX_form.html). For phylogenetic analysis and PTS1 conservation, and species abbreviations and numbers, see also Fig.1
