# Supplementary material for: Multi-targeted trehalose-6-phosphate phosphatase I harbors a novel peroxisomal targeting signal 1 and is essential for flowering and development
Source: Planta. 2020 Apr 18;251(5):98. doi: 10.1007/s00425-020-03389-z (PMC7214503; doi:10.1007/s00425-020-03389-z)
Supplement: Supplementary file 2 — Supplementary file2 (PDF 195 kb) [file 425_2020_3389_MOESM2_ESM.pdf]

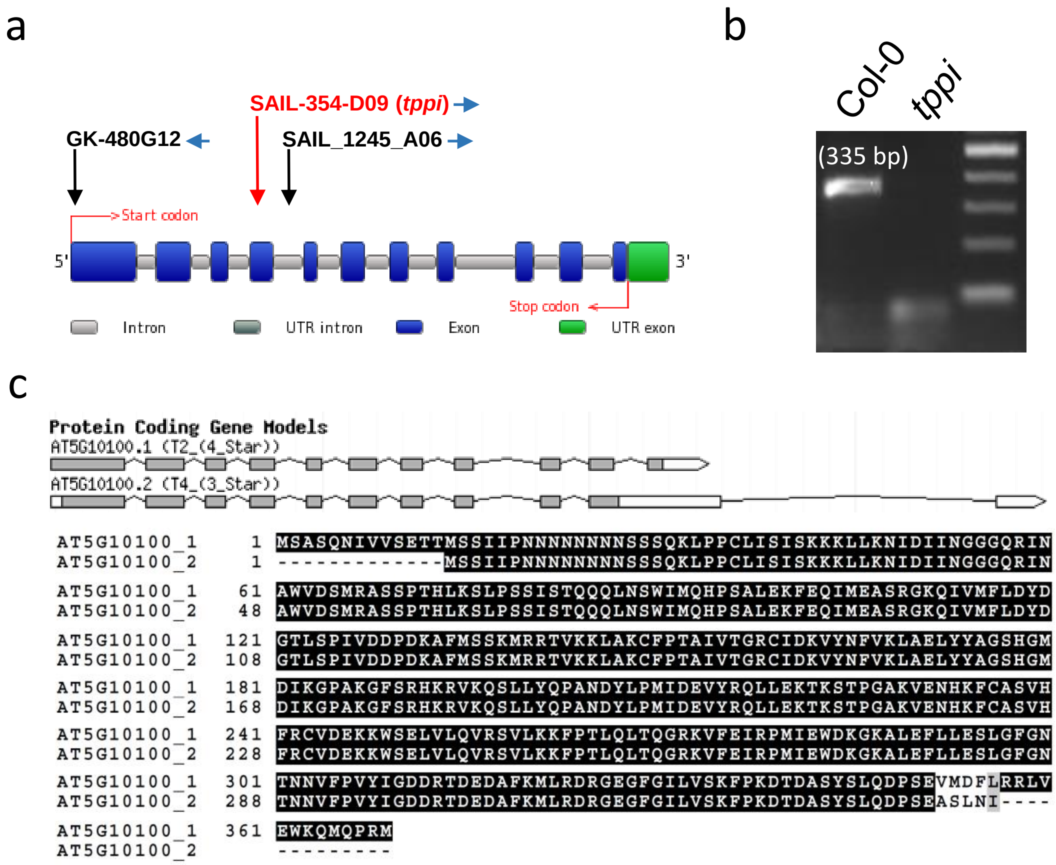


**Supplementary Fig. S2** Molecular characterization of *TPPI* knockout mutant. **a** Schematic presentation of T-DNA insertion lines of the newly identified peroxisomal TPPI. The scheme was taken from the PLAZA database (Van Bel et al. 2012) and was modified by highlighting T-DNA insertions. **b** Reverse transcription-polymerase chain reaction (RT-PCR) analysis using primers (forward that is spanning exons 2 and 3, and reverse that binds in exon 6). The TPPI was found to be knocked out in the *tppi* mutant. **c** Schematic representation of the *TPPI* gene and their splice variants uploaded from the TAIR website, and the variants CDS were aligned by ClustalW/MEGA6 (Tamura et al. 2013), and conserved residues were shaded by BoxShade (http://www.ch.embnet.org/software/BOX_form.html). The alignments show that the peroxisomal signal (PRM>) and the strong chloroplast signal (N-9 aa) are only present in variant 1
