# Supplementary material for: Multi-targeted trehalose-6-phosphate phosphatase I harbors a novel peroxisomal targeting signal 1 and is essential for flowering and development
Source: Planta. 2020 Apr 18;251(5):98. doi: 10.1007/s00425-020-03389-z (PMC7214503; doi:10.1007/s00425-020-03389-z)
Supplement: Supplementary file 3 — Supplementary file3 (PDF 239 kb) [file 425_2020_3389_MOESM3_ESM.pdf]

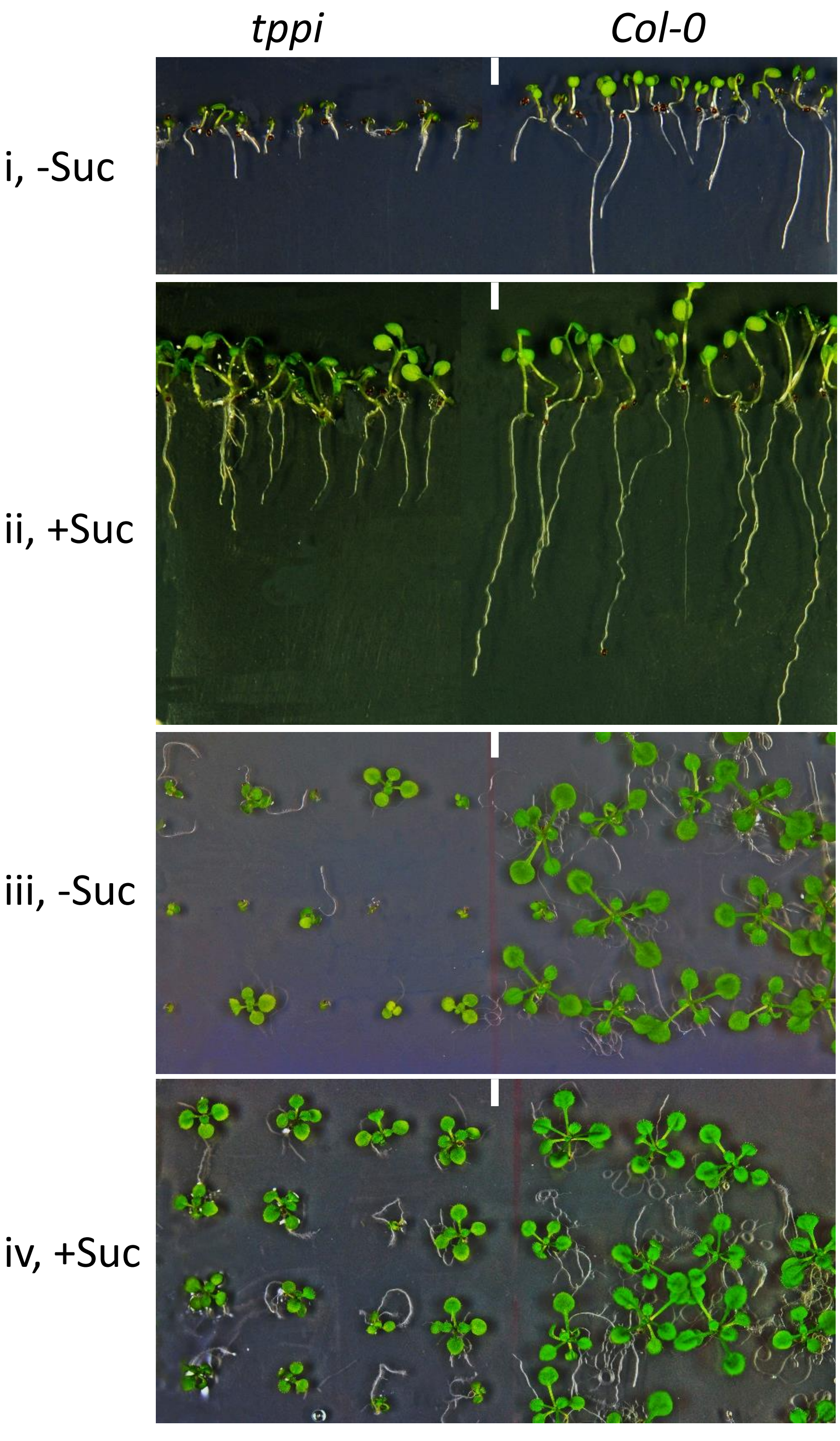

Supplementary Fig. S3 Sucrose dependence of *tppi* mutant in short and long-day conditions. Seedlings were grown for 6 d (**i**, **ii)** in 8 h of light/ 16 h of dark, and for 14 d (**iii**, **iv**) in 16 h of light/ 8 h of dark on one-half-strength Linsmeier and Skoog (LS) medium with or without 1% Sucrose
