# Supplementary material for: Multi-targeted trehalose-6-phosphate phosphatase I harbors a novel peroxisomal targeting signal 1 and is essential for flowering and development
Source: Planta. 2020 Apr 18;251(5):98. doi: 10.1007/s00425-020-03389-z (PMC7214503; doi:10.1007/s00425-020-03389-z)
Supplement: Supplementary file 4 — Supplementary file4 (PDF 215 kb) [file 425_2020_3389_MOESM4_ESM.pdf]

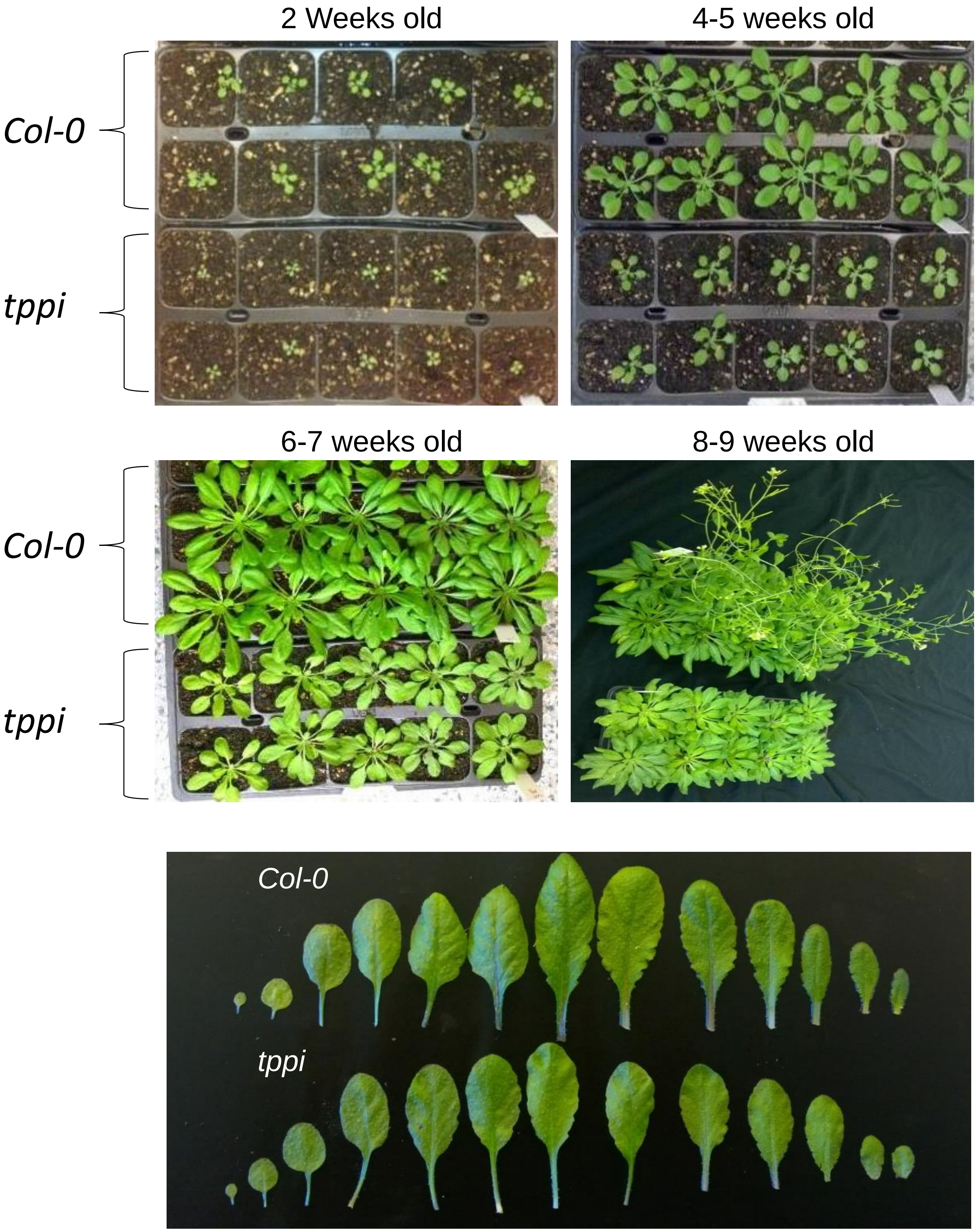

Supplementary Fig. S4 Rosette stage plants grown in the soil also showed impaired growth, and they flowered much later
