# Supplementary material for: Multi-targeted trehalose-6-phosphate phosphatase I harbors a novel peroxisomal targeting signal 1 and is essential for flowering and development
Source: Planta. 2020 Apr 18;251(5):98. doi: 10.1007/s00425-020-03389-z (PMC7214503; doi:10.1007/s00425-020-03389-z)
Supplement: Supplementary file 5 — Supplementary file5 (PDF 144 kb) [file 425_2020_3389_MOESM5_ESM.pdf]

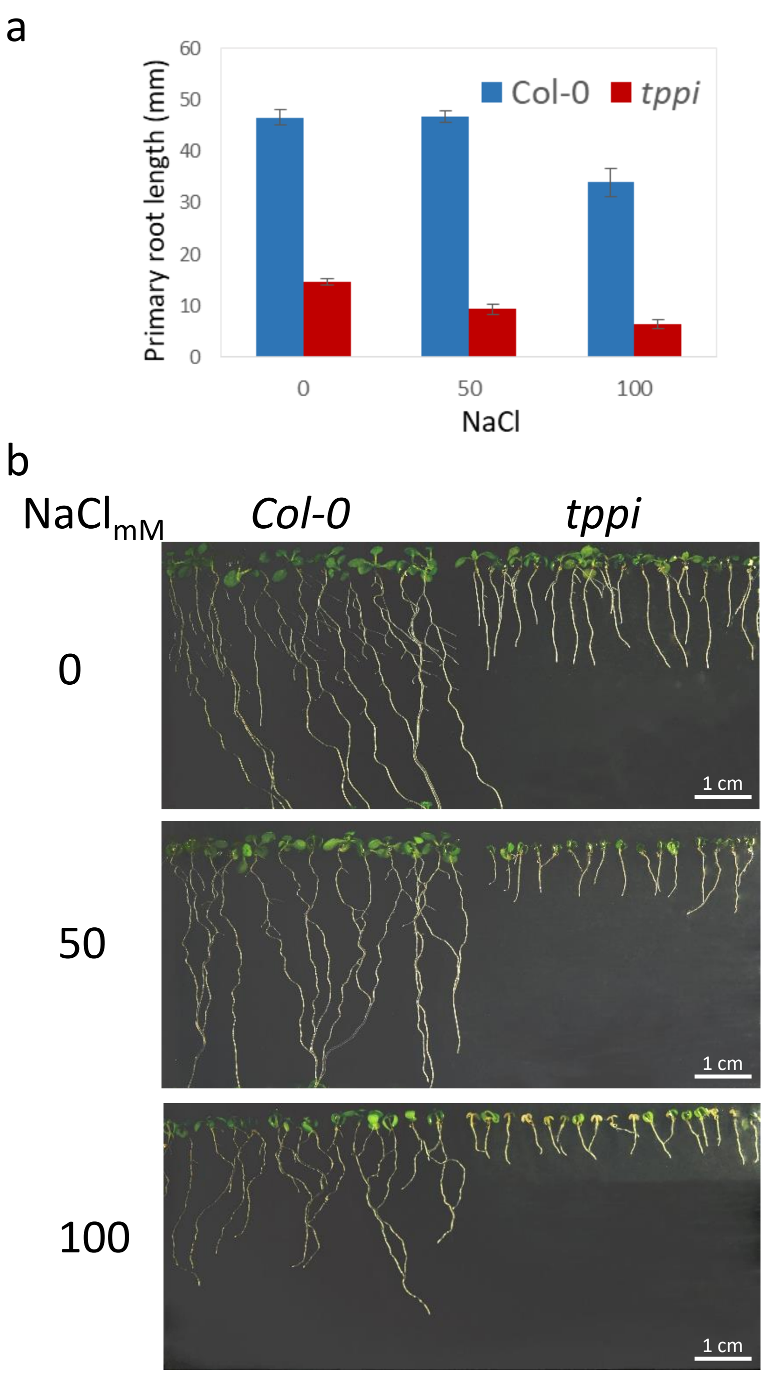

Supplementary Fig. S5 Hypersensitivity of *tppi* mutant seedlings to salt stress. WT and *tppi* mutant seeds were sown and grown for 4 d in 16 h of light/ 8 h of dark on one-half-strength Linsmeier and Skoog (LS) medium with 1% Suc. The seedlings were then transferred to the same media containing the indicated NaCl amounts and allowed to grow under the same conditions for another 6 days. **a** Effects of salt stress on primary root elongation of *tppi* mutant seedlings. Root lengths of 10-d-old seedlings were measured by the ImageJ program. The experiments were repeated three times; error bars represent SE. **b** Images representing 10-d-old seedlings that were allowed to grow on salt-free media, and media with 50 mM and 100 mM NaCl. These images show clearly that *tppi* seedlings are hypersensitive to salt when compared to WT
