# Supplementary material for: Multi-targeted trehalose-6-phosphate phosphatase I harbors a novel peroxisomal targeting signal 1 and is essential for flowering and development
Source: Planta. 2020 Apr 18;251(5):98. doi: 10.1007/s00425-020-03389-z (PMC7214503; doi:10.1007/s00425-020-03389-z)
Supplement: Supplementary file 6 — Supplementary file6 (PDF 188 kb) [file 425_2020_3389_MOESM6_ESM.pdf]

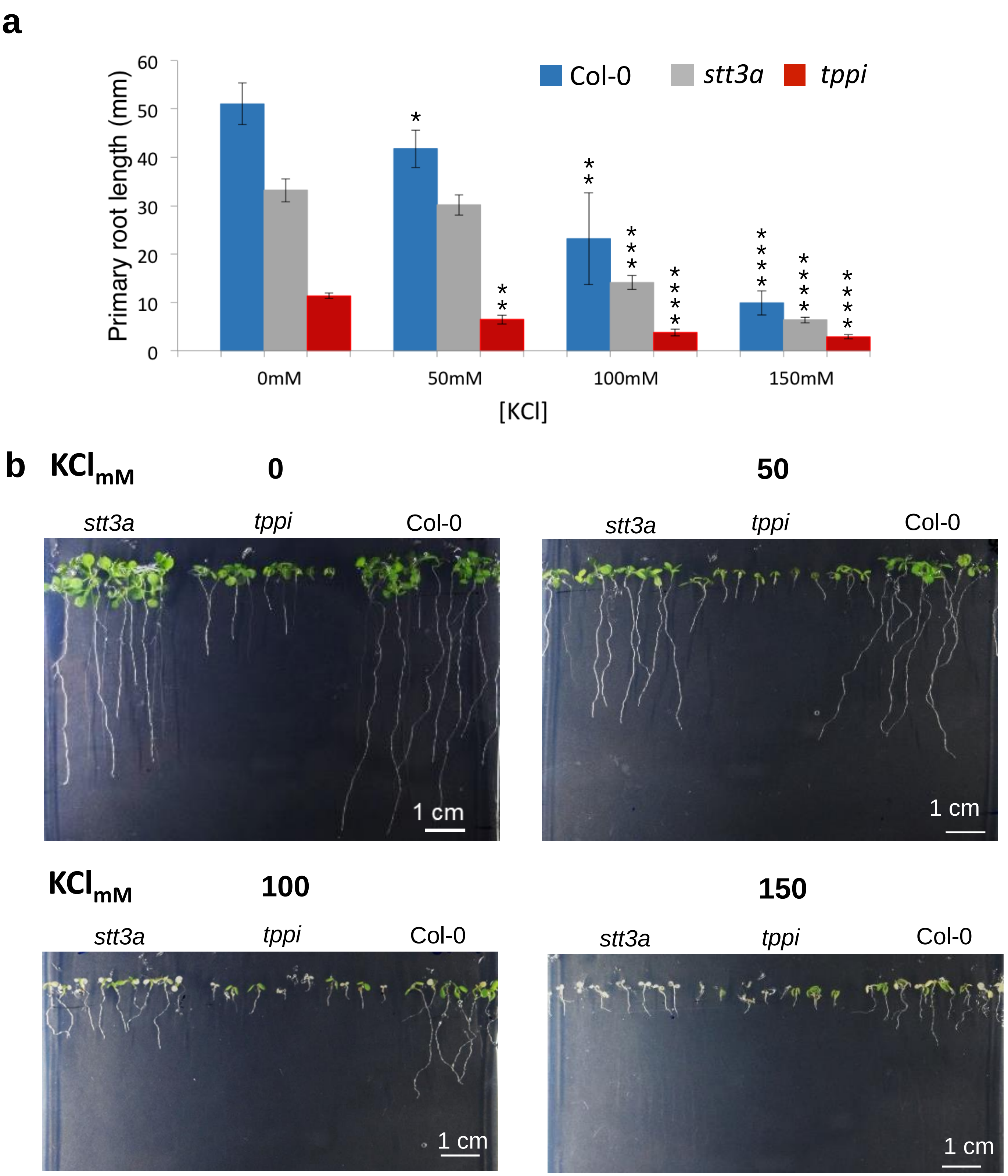

Supplementary Fig. S6 The *tppi* mutant is hypersensitive to KCl stress. **a** The *tppi* mutant shows reduced root growth upon exposure to KCl stress. Eight roots were measured per genotype, and the experiment was repeated three times. * $P < 0.05$, ** $P < 0.01$, *** $P < 0.001$ and **** $P < 0.0001$. Two-tailed t-test with equal variance (relative to 0 mM KCl). Error bars indicate standard deviation. **b** Root growth of representative seedlings of WT (Col-0), *tppi*, and *stt3a* grown on LS agar containing 0, 50, 100, or 150 mM KCl. Some *tppi* individuals died at 150 mM KCl. *STT3a* encodes a subunit of oligosaccharyltransferase complex involved in response to high salt conditions, and *stt3a* is hypersensitive to high molarities of KCl (Koiwa et al. 2003)
