# Supplementary material for: Multi-targeted trehalose-6-phosphate phosphatase I harbors a novel peroxisomal targeting signal 1 and is essential for flowering and development
Source: Planta. 2020 Apr 18;251(5):98. doi: 10.1007/s00425-020-03389-z (PMC7214503; doi:10.1007/s00425-020-03389-z)
Supplement: Supplementary file 7 — Supplementary file7 (PDF 55 kb) [file 425_2020_3389_MOESM7_ESM.pdf]

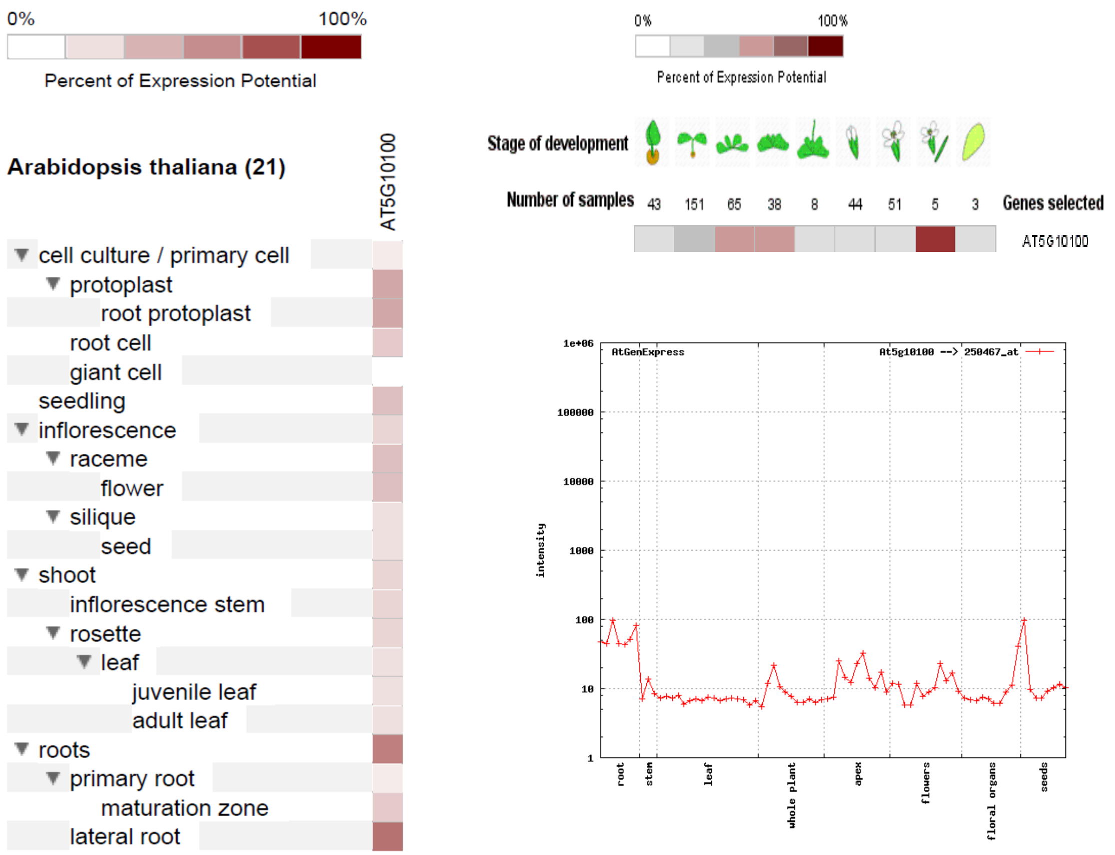

Supplementary Fig. S7 *TPPI* is highly expressed in roots and flowers by Genevestigator (https://genevestigator.com/gv/)
